# Aβ/tau oligomer interplay at human synapses supports shifting therapeutic targets for Alzheimer’s disease

**DOI:** 10.1007/s00018-022-04255-9

**Published:** 2022-04-04

**Authors:** Michela Marcatti, Anna Fracassi, Mauro Montalbano, Chandramouli Natarajan, Balaji Krishnan, Rakez Kayed, Giulio Taglialatela

**Affiliations:** Department of Neurology, Mitchell Center for Neurodegenerative Diseases, University of Texas Medical Branch, Galveston, TX 77555 USA

**Keywords:** Amyloid, Tau, Synaptic binding, Synaptosomes, Dementia

## Abstract

**Background:**

Alzheimer’s disease (AD) is characterized by progressive cognitive decline due to accumulating synaptic insults by toxic oligomers of amyloid beta (AβO) and tau (TauO). There is growing consensus that preventing these oligomers from interacting with synapses might be an effective approach to treat AD. However, recent clinical trial failures suggest low effectiveness of targeting Aβ in late-stage AD. Researchers have redirected their attention toward TauO as the levels of this species increase later in disease pathogenesis. Here we show that AβO and TauO differentially target synapses and affect each other's binding dynamics.

**Methods:**

Binding of labeled, pre-formed Aβ and tau oligomers onto synaptosomes isolated from the hippocampus and frontal cortex of mouse and *postmortem* cognitively intact elderly human brains was evaluated using flow-cytometry and western blot analyses. Binding of labeled, pre-formed Aβ and tau oligomers onto mouse primary neurons was assessed using immunofluorescence assay. The synaptic dysfunction was measured by fluorescence analysis of single-synapse long-term potentiation (FASS-LTP) assay.

**Results:**

We demonstrated that higher TauO concentrations effectively outcompete AβO and become the prevailing synaptic-associated species. Conversely, high concentrations of AβO facilitate synaptic TauO recruitment. Immunofluorescence analyses of mouse primary cortical neurons confirmed differential synaptic binding dynamics of AβO and TauO. Moreover, in vivo experiments using old 3xTgAD mice ICV injected with either AβO or TauO fully supported these findings. Consistent with these observations, FASS-LTP analyses demonstrated that TauO-induced suppression of chemical LTP was exacerbated by AβO. Finally, predigestion with proteinase K abolished the ability of TauO to compete off AβO without affecting the ability of high AβO levels to increase synaptic TauO recruitment. Thus, unlike AβO, TauO effects on synaptosomes are hampered by the absence of protein substrate in the membrane.

**Conclusions:**

These results introduce the concept that TauO become the main synaptotoxic species at late AD, thus supporting the hypothesis that TauO may be the most effective therapeutic target for clinically manifest AD.

**Supplementary Information:**

The online version contains supplementary material available at 10.1007/s00018-022-04255-9.

## Introduction

Alzheimer’s disease (AD) is the most common age-related neurodegenerative disorder, clinically characterized by progressive cognitive decline and memory dysfunction [1]. The histopathologic hallmarks of AD are extracellular plaques of insoluble fibrillar aggregates of the amyloid beta peptide (Aβ) and intracellular neurofibrillary tangles of hyperphosphorylated tau protein [2]. These large aggregates are formed through a process of protein misfolding, aggregation, and deposition that begins with the formation of small soluble oligomers, now thought to be the most toxic species of both Aβ and tau [3, 4]. Compelling evidence supports the hypothesis that soluble oligomers of both AβO and tau (TauO) can target synapses and induce their dysfunction to cause the clinical onset and progression of AD [3, 5, 6]. Both AβO and TauO have been found in synaptic terminals (synaptosomes) isolated from AD brains [7, 8]. These findings spurred the concept of AD as a synaptic disease [9, 10]. Numerous in vitro and in vivo studies reported detrimental impacts of AβO and TauO on synaptic plasticity and memory formation, even before the overt appearance of Aβ plaques or tau tangles [11–15]. The mechanisms underlying these phenomena remain poorly understood. Notably, while both AβO and TauO can individually affect synaptic function, they can also act synergistically. Low concentrations of AβO and TauO that would not normally perturb synaptic function effectively suppress long-term potentiation (LTP) and impair memory function when administered together [13]. The colocalization of AβO and TauO at the synaptosomes isolated from AD brains has been observed [7]. Some studies showed that AβO and TauO impair synaptic plasticity and memory independently [16, 17], while others reported that AβO acts upstream of TauO to drive AD pathogenesis [8, 18, 19].

Synaptic dysfunction and associated memory deficits are important contributors to dementia in AD, and therapeutic approaches to prevent these deficits have the potential to prevent or reverse progression of cognitive decline. Recent partial failures of Aβ-directed therapeutics in multi-center clinical trials [2] extended the interest onto tau, which may be the primary synaptotoxic element during late stages of AD. Attention is currently focused on the development of tau-directed therapies for clinical AD [3, 20, 21], but the mechanisms by which tau may drive synaptic dysfunction at later disease stages remain unclear. With all this evidence in mind, here we investigated the synaptic binding dynamics of both AβO and TauO using synaptosomes isolated from human and wild type mouse brains, wild type mouse brain slices, mouse cortical primary neurons, and 3xTgAD brains and report that TauO can outcompete and supersede AβO at the synapse. This suggests that, as the disease progresses TauO accumulate in the brain overcoming AβO at the synapse, thus becoming the most prevalent and toxic species supporting the concept of tau as an important therapeutic target [20, 21].

## Methods

### Human subjects and autopsy of brain tissues

*Postmortem* frozen brain tissues were obtained from the Oregon Brain Bank at Oregon Health and Science University (OHSU; Portland, OR, USA). Donor subjects of either sex were enrolled and clinically evaluated in studies at the National Institutes of Health (NIH)-sponsored Layton Aging and AD Center (ADC) at OHSU, in accordance with protocols approved by the OHSU Institutional Review Board (IRB). Informed consent was obtained from all participants prior to their enrolment in brain aging studies at the ADC; each subject received annual neurological and neuropsychological evaluations, with a Clinical Dementia Rating assigned by an experienced clinician. A neuropathological assessment was performed at autopsy in compliance with IRB-approved protocols. A neuropathologist scored autopsy brain tissue for Aβ plaques and neurofibrillary tangles according to standardized CERAD (Consortium to Establish a Registry for AD) criteria and Braak staging [22]. The tissues used in this study were from subjects classified as controls, because they had normal cognitive examination results (Mini-Mental State Examination scores 0–30) [23]. Donor subject samples were de-identified by the ADC prior to shipment to the University of Texas Medical Branch (UTMB), so no approval was required from the UTMB IRB under CFR §46.101(a)(1). The cases used in this study are described in Supplementary Table 1 (Additional file 1).

### Animals

Wild-type 11–13-week-old male and female mice (C57BL/6J *Mus musculus*-Cat# JAX:000664, RRID: IMSR JAX:000664) and 9 male 3xTgAD mice (B6.Cg–Tg (APPSwe,tauP301L)1Lfa Psen1tm1Mpm/2J *Mus musculus*-Cat# JAX: 033930) were purchased from the Jackson Laboratory (Bar Harbor, ME, USA). Health care was provided by the animal care specialists under supervision of the facility manager. Animal colony care and maintenance were provided daily to ensure a safe, healthy environment. Each animal was used under a protocol approved by UTMB’s Institutional Animal Care and Use Committee, ensuring that the animals experienced the minimal amount of pain/discomfort. All animals were housed under USDA standards (12:12-h light/dark cycle, ad libitum food and water) at the UTMB vivarium.

### Synaptosome isolation

The synaptosomal fraction containing both pre- and postsynaptic components was isolated using a well-established method developed in our laboratory [24–26]. Briefly, we lysed snap frozen hippocampus (HP) and frontal cortex (FC) tissue from mouse and cognitively intact elderly human brains using SynPER lysis buffer (Thermo Fisher Scientific, Waltham, MA, USA) with 1% protease and phosphatase cocktail inhibitors. The brain homogenates were centrifuged at 1200 × *g* for 10 min at 4 °C. The supernatants (containing the synaptosomes) were collected and centrifuged at 15,000 × *g* for 20 min at 4 °C. The synaptosomal pellets were resuspended in HEPES-buffered Krebs-like (HBK) buffer (143.3 mM NaCl, 4.75 mM KCl, 1.2 mM MgSO_4_·7H_2_O, 1.2 mM CaCl_2_, 20.1 mM HEPES, 0.1 mM NaH_2_PO_4_, and 10.3 mM d-glucose, pH 7.4). Finally, 0.5% of Pluronic F-68 non-ionic surfactant (cat# 24040-032, lot# 2275337; Thermo Fisher Scientific) was added to prevent synaptosome aggregation as previously described [27]. The quality and concentration (synaptosomes/µl) of isolated synaptosomes was routinely verified by flow cytometry and electron microscopy as we previously reported [24]. Sample processing for the ultrastructure, flow cytometry, and protein analyses are described in detail in the Supplementary Methods (Additional file 1).

### AβO preparation

Human Aβ1–42 peptide was purchased from Department of Biophysics and Biochemistry, Harvard University (Cambridge, MA, USA), and AβO were prepared from lyophilized synthetic Aβ aliquots as previously described [28]. Briefly, 0.3 mg of lyophilized Aβ were dissolved in 200 µl of 1,1,1,3,3,3-hexafluoro-2-propanol (HFP) and allowed to incubate at room temperature for 10–20 min. Then, 700 µl of double-deionized water was added, and the sample was magnetically stirred in a fume hood for 48 h at room temperature (RT). A cap with four holes was placed on the tube containing the sample to allow HFP evaporation. The obtained AβO were aliquoted, frozen at − 80 °C, and used within 3 months of preparation. Flow cytometry analysis of AβO binding to synaptosomes was performed using AβO spiked with HyLite Fluor 647-tagged Aβ (cat# AS-64161; AnaSpec Inc., Fremont, CA, USA). These AβO (AβO647) were prepared by adding 7 µl of tagged Aβ to the HFP-Aβ mixture prior to AβO formation. Oligomeric preparation quality was checked by western blot analysis using the AβO-specific 6E10 antibody (cat# 803002; BioLegend, San Diego, Ca, USA). AβO have been well characterized by our group and others [10, 29–32].

### TauO preparation

Prepared recombinant TauO were provided by Dr. Rakez Kayed’s laboratory. They were produced and characterized following established and published protocols [33, 34] and labeled (TauO488) as previously described [35–37]. Briefly, 1 mg of Alexa Fluor™ 488 NHS Ester Succinimidyl Ester (cat# A20000, Thermo Fisher Scientific) was dissolved in 0.1 M sodium bicarbonate to a final concentration 1 mg/ml, pH 8.3. The dye was then incubated with TauO in a 1:4 (w/w) ratio, rotating overnight at 4 °C on an orbital shaker. The following day, the solution was centrifuged (30 min, 15,000 × *g*) using 10-kDa Amicon Ultra‐0.5 ml Centrifugal Filter Units (cat# UFC501024; EMD Millipore, Burlington, MA, USA) to remove unbound dye. TauO were then washed with 1 × phosphate-buffered saline (PBS) until the flow-through solution was clear. The filter compartment was then flipped and centrifuged to collect the concentrate (2 min, 1000 × *g*). Oligomer concentrations were then quantified with the Pierce™ BCA Protein Assay Kit (cat# 23227, Thermo Fisher Scientific) and used for flow cytometry and immunofluorescence analyses.

### α-Sinuclein oligomer (α-synO) preparation

Prepared recombinant α-synO were provided by Dr. Rakez Kayed’s laboratory. They were produced and characterized following established and published protocols [38, 39]. Briefly, an aliquot of lyophilized α-syn protein was dissolved in 280 μl of 1,1,1,3,3,3-hexafluoro-2-propanol (HFP) and allowed to incubate at room temperature (RT) for 10–20 min. Double-deionized water was added to this solution to make the final concentration 0.7 μg/μl. The resulting solution was then magnetically stirred in a fume hood for 48 h inside the fume hood at RT closed with a cap with holes to allow the evaporation of HFP.

### AβO and TauO binding challenge to synaptosomes

Synaptosomes were treated with AβO and/or TauO for binding challenges, and the binding percentages were evaluated with flow cytometry. After assessing that the binding of AβO and/or TauO was comparable among the single human cases (Supplementary method 2), we decided to pool together an equal number of synaptosomes isolated from each subject for practical purpose. We incubated 2 million of synaptosomes for 1 h at RT without oligomers (control) as well as with AβO tagged with HyLite Fluor 647 or/and TauO tagged with Alexa Fluor™ 488 NHS Ester (Thermo Fisher Scientific) at concentrations of 0–0.5–1–2.5–5–10 μM. Synaptosomes were then pelleted, washed three times with HBK buffer, and resuspended in HBK. Oligomer fluorescence positivity was acquired by a Guava EasyCyte 8 flow cytometer (EMD Millipore) and analyzed using Incyte software (EMD Millipore).

### AβO and Tau oligomers binding challenge to mouse brain slices

For this set of experiments, 3–4-month-old C57BL/6J mice were euthanized with deep isoflurane anesthesia followed by cervical dislocation, and the brains were immediately collected and sliced using a Compresstome VF-300 (Precisionary Instruments, Greenville, NC, USA) in *N*-methyl-d-glucamine–artificial cerebrospinal fluid (NMDG–aCSF) buffer (93 mM NMDG, 2.5 mM KCl, 1.2 mM NaH_2_PO_4_, 30 mM NaHCO_3_, 20 mM HEPES, 25 mM glucose, 5 M sodium ascorbate, 2 mM thiourea, 3 mM sodium pyruvate, 10 mM MgSO_4_·7H_2_O, 0.5 mM CaCl_2_ 2H_2_O, and 12 mM *N*-acetyl l-Cysteine) to obtain 350-μm horizontal brain sections. Slices were allowed to recover for 30 min in NMDG–aCSF–NaCl buffer at 33 °C. Slices were then maintained at RT in a modified HEPES holding–aCSF solution (92 mM NaCl, 2.5 mM KCl, 1.2 mM NaH_2_PO_4_, 30 mM NaHCO_3_, 20 mM HEPES, 25 mM glucose, 5 mM sodium ascorbate, 2 mM thiourea, 3 mM sodium pyruvate, 2 mM MgSO_4_ 7H_2_O, 2 mM CaCl_2_ 2H_2_O, 12 *N*-Acetyl l-Cysteine). For oligomer challenges, the slices were incubated for 1 h at RT with AβO and/or TauO at concentrations of 0–0.05–0.5–1–2.5 μM. After treatment, synaptosomes were isolated, pelleted, washed three times with HBK buffer, and resuspended in HBK buffer. Oligomer fluorescence positivity of synaptosomes was measured as described above.

### Proteinase K digestion

Following the challenge experiments, synaptosomes were digested with 1 mg/ml of proteinase K (PK; cat# 70663-4, lot# 3018798; EMD Millipore) for 30 min at 37 °C (1 mg of PK is the equivalent of 30 mAU, where AU is an Anson unit that represents the amount of enzyme that liberates 1.0 µmol (181 µg) of tyrosine from casein per min at pH 7.5 at 37 °C). The remaining oligomer binding positivity was measured by a Guava EasyCyte flow cytometer (EMD Millipore) and analyzed using Incyte software (EMD Millipore). For pretreatment experiments, synaptosomes were digested with 1 mg/ml of PK (cat# 70663-4, lot# 3018798; EMD Millipore) for 30 min at 37 °C prior to challenge with labeled AβO and TauO. Binding was measured as for the other experiments.

### Western blot

Synaptosomes were treated with AβO and/or TauO for binding challenges, and the binding percentages were evaluated by western blotting analyses. We pooled together an equal number of synaptosomes isolated from each sample. We incubated 2 million of synaptosomes for 1 h at RT without oligomers (control) as well as with AβO and TauO (prepared as above) at the concentrations of 0–2.5–10 μM. Synaptosomes were washed three times with HBK buffer, and the pellets were lysed with 1 × radioimmunoprecipitation assay buffer (RIPA buffer) with 1% protease and phosphatase cocktail inhibitors. Tricine sample buffer (Thermo Fisher Scientific) was added to the total proteins derived from AβO-treated synaptosomes to a final concentration of 1 × . Then, proteins were loaded in a 16% Novex Tricine gels (Thermo Fisher Scientific) followed by 45 min transfer to Amersham Protran nitrocellulose transfer membranes (GE Healthcare-Life Sciences, Chicago, IL, USA) at 85 V at 4 °C. The use of tricine sample buffer and gel is well recommended for the detection of low molecular weight proteins. Lithium dodecyl sulfate sample buffer (Thermo Fisher Scientific) was added to the total proteins derived from TauO-treated synaptosomes to a final concentration of 1 × . Then, proteins were loaded in a 12% NuPAGE Bis–Tris gels (Thermo Fisher Scientific) followed by 1 h transfer to Amersham Protran nitrocellulose transfer membranes (GE Healthcare-Life Sciences) at 95 V at 4 °C. The membranes were blocked using Odyssey blocking buffer (LI-COR, Lincoln, NE, USA) for 1 h at RT and incubated at 4 °C overnight with the anti-Aβ antibody (6E10, cat# 803002; RRID:AB_2564654; 1:1000 dilution; BioLegend) or the anti-tau antibody (Tau5; cat# 806402; RRID:AB_2564706; 1:1000 dilution; BioLegend) and 1 h at RT with the anti-synaptophysin (SYPH) antibody (cat# ab8049; RRID:AB_2198854; 1:10,000 diluted; Abcam, Cambridge, UK). All primary antibodies were prepared in a 1:1 solution of 1 × Tris-buffered saline solution with Tween (TBST) and Odyssey blocking buffer. After incubation, the membranes were washed three times with 1 × TBST (10 min each) and incubated 1 h with LI-COR secondary antibodies diluted at 1:10,000 in 1 × TBST-Odyssey blocking buffer at RT. The membranes were again washed three times for 10 min each. Western blots were imaged using an LI-COR Odyssey infrared imaging system, application software version 3.0.30. The density of immunoreactive oligomeric bands (selected all together) were measured using ImageJ FIJI software (https://imagej.nih.gov/ij, NIH, Bethesda, MD, USA).

### Primary neuron isolation

Primary cortical neuronal cultures were prepared and maintained as described previously [37]. Briefly, cortical neurons were isolated from C57BL/6 mice during embryonic days 16–18 by gentle trituration by a fire-polished glass pasteur pipet with Accutase^®^ solution (cat# A6964, Sigma, St. Louis, MO, USA). Dissociated cells were plated at a density of 5 × 10^5^ cells/ml in Ibidi µ-Slide 8 Well Glass Bottom (cat# 80827; Ibidi GmbH, Martinsried, Germany) containing high-glucose Dulbecco’s Modified Eagle’s Medium (cat# 10–013-CV; Corning, Corning, NY, USA) with 2% B-27 Plus supplement (cat# A3582801; Gibco/Thermo Fisher Scientific), 10,000 U/ml penicillin, 10,000 μg/ml streptomycin, and 25 μg/ml amphotericin B (cat# 15240062, Gibco/Thermo Fisher Scientific). After 2 h, plating medium was removed from ls and replenished with neurobasal medium (cat# 12348017, Gibco/Thermo Fisher Scientific) plus 2% B-27 Plus, 0.5 mM GlutaMax (cat# 35050-061, Gibco/Thermo Fisher Scientific), 10,000 U/ml penicillin, 10,000 μg/ml streptomycin, and 25 μg/ml amphotericin B supplement. Half of the medium was changed every 3–5 days. Cells on days 7–10 in vitro were used for all experiments.

### Primary neuron treatment and immunofluorescence

Cultured neurons were exposed to AβO647 and TauO488 at concentrations of 2.5 μM at 37 °C for 30 min. After treatment, oligomer-containing media were removed, and cells were washed three times with 1 × PBS (5 min each). Cells were fixed with 300 μl of 4% paraformaldehyde (PFA)/PBS for 15 min at RT. Cells were then permeabilized in 300 μl of 0.25% Triton X‐100 in PBS (PBST) for 10 min at RT and then washed three times in 1 × PBS (5 min each). Blocking was done in 300 μl of 5% normal goat serum/5% bovine serum albumin (BSA) in PBST for 1.5–2 h. Primary antibodies were diluted 1:500 in 5% BSA/PBST for overnight incubation at 4 °C (microtubule-associated protein 2 [MAP2] antibody, cat# MAP2; Aves Labs, Tigard, OR, USA; postsynaptic density-95 [PSD95] antibody, cat# ab13552, RRID:AB 300453, Abcam). After primary antibody incubation, the cells were washed three times in PBST (10 min each). Secondary antibodies from Thermo Fisher Scientific were diluted in 5% BSA/PBST for 2 h at RT (Goat anti-Rat IgG (H + L) Cross-Adsorbed Secondary Antibody, Alexa Fluor 594, cat# A11007, RRID:AB_141374; 1:400 diluted; Goat anti-Chicken IgY (H + L) Cross-Adsorbed Secondary Antibody, Alexa Fluor Plus 405, cat# A48260; 1:250 diluted). Total number of PSD95 puncta has been calculated using ImageJ FIJI (NIH) Software with analyze particle plugin after thresholding neuronal projections 30 µm in length (*n* = 18 per group). Synaptic AβO and TauO has been evaluated as oligomers co-localizing with PSD95 puncta. Using colocalization threshold plugin function in ImageJ FIJI we measured the synaptic density of AβO and TauO co-localizing with PSD95. The grade of synapses/oligomers association, in each study group, has been calculated with the following ratio: colocalizing AβO/PSD95 puncta divided by the total number of PSD95 puncta in the same projection. Same method has been used for synaptic TauO in neuronal projections.

### Intracerebroventricular (ICV) injections

16-month-old 3xTgAD mice were anesthetized with isoflurane and subjected to ICV injections using the freehand injection method previously described [40, 41]. Briefly, a 29-gauge needle, firmly held with hemostatic forceps to leave 4.5 mm of the needle tip exposed, was connected to a 25 μl Hamilton syringe via 0.38 mm polyethylene tubing. Infusions were performed at the rate of 3 μl/min for a total volume of 3 μl, using an electronic programmable microinfuser (Harvard Apparatus). Mice were ICV injected with 3ul of 0.55 µM of either AβO or TauO. After ICV injection, the needle was left in place for 2 min, while the mouse was allowed to recover lying on a heated pad under warm light. 24 h after ICV injection of oligomers or PBS, mice were euthanized and the brains were removed, dissected, snap frozen on dry ice, and stored at − 80 °C. Then, we isolated synaptosomes from hippocampus and frontal cortex and subjected the protein extracts to western blot analysis to evaluate the effects of the ICV injected AβO and TauO on endogenous TauO and AβO, respectively, in comparison with the control mice (ICV with PBS).

### Fluorescence-assisted single synaptosome long-term potentiation

Fluorescence-assisted single synaptosome long-term potentiation (FASS-LTP) is a chemically induced LTP technique (cLTP) focused on the insertion of the glutamatergic α-amino-3-hydroxy-5-methyl-4-isoxazolepropionic acid receptor (AMPAR) GluA1 into the postsynaptic surface, which is an essential event for synaptic transmission potentiation. FASS-LTP identifies potentiated synapses by tracking the surface expression in size-gated, glycine-activated synaptosomes of GluA1 and neurexin-1β (Nrx1β), a presynaptic adhesion molecule stabilized at the membrane surface by synaptic activity. FASS-LTP uses antibodies specific for extracellular epitopes on GluA1 and Nrx1β and we measured GluR1 + Nrx1β + double-labeled synaptosomes to ensures the analysis of only intact synaptosomes that contain both presynaptic and postsynaptic elements. FASS-LTP experiments were conducted as previously described [42–44]. Briefly, 5 × 10^6^ synaptosomes were suspended in separate tubes containing different solutions. Tube E contained 200 µl of extracellular solution (20 mM NaCl, 3 mM KCl, 2 mM CaCl_2_, 2 mM MgCl_2_, 15 mM glucose, and 15 mM HEPES, pH 7.4), tube B contained 200 µl of basal solution (20 mM NaCl, 3 mM KCl, 2 mM CaCl_2_, 2 mM MgCl_2_, 15 mM glucose, and 15 mM HEPES, pH 7.4), and tube C contained 200 µl of cLTP solution (150 mM NaCl, 5 mM KCl, 2 mM CaCl_2_, 30 mM glucose, and 10 mM HEPES, pH 7.4). Synaptosomes in extracellular solution were used to determine the basal levels of potentiated synaptosomes. All tubes were incubated at RT on a slowly oscillating shaker to thaw the frozen samples. For stimulation, 20 µl of 5 mM glycine (*N*-methyl d-aspartate receptor [NMDAR] co-agonist) was added to the tube C freshly supplemented with 0.001 mM strychnine and 0.02 mM bicuculline methiodide. Equivalent amounts of extracellular solution were added to control tubes E and B. Following stimulation, synaptosomes in tube C were depolarized with 100 µl of KCl solution (50 mM NaCl, 100 mM KCl, 2 mM CaCl_2_, 30 mM glucose, 10 mM HEPES, 0.5 mM glycine, 0.001 mM strychnine, 0.02 mM bicuculline) and incubated at 37 °C for 30 min. This step is based on the principle that high KCl concentrations depolarize synaptosomes to release endogenous glutamate, which further activates synaptic NMDARs in conjunction with the co-agonist glycine. Equivalent amounts of extracellular solution were added to tubes E and B and incubated along with tube C for 30 min at 37 °C. The contents in tubes E, B, and C were transferred to 15-ml centrifuge tubes. Then, 0.5 ml of ice-cold 0.1 mM EDTA–PBS solution and 4 ml of 5% blocking buffer (5% fetal bovine serum in PBS) were added to tubes E, B, and C to stop the reaction. Tubes were kept on ice and centrifuged at 2500 × *g* for 5 min at 4 °C, and the supernatant was discarded. The translocated AMPARs are then captured by adding to the tubes B and C, 2.5 µg/ml of the primary antibodies specific for the extracellular epitopes GluR1 (anti-GluR1 antibody, cat# ABN241, RRID:AB_2721164, EMD Millipore) and Nrx1β (cat#75-216, RRID:AB_2155531; Antibodies Incorporated, Davis, CA, USA) prepared in blocking solution. After incubation with the primary antibodies and subsequent washes with 1 × PBS, the samples were centrifuged at 2500 × *g* for 5 min at 4 °C. Pellets were resuspended in 100 µl of secondary antibody solution (Goat anti-Mouse IgG (H + L) Highly Cross-Adsorbed Secondary Antibody, Alexa Fluor 647, cat#A-21236, RRID:AB_2535805; Goat anti-Rabbit IgG (H + L) Highly Cross-Adsorbed Secondary Antibody, AlexaFluor 488, cat#A-11034, RRID:AB_2576217; both from Thermo Fisher Scientific). After 45 min of incubation at 37 °C, synaptosomes were washed twice with 1 × PBS and then centrifuged at 2500 × *g* for 5 min. Endogenous/nonspecific background fluorescence for each marker was determined using secondary antibody staining only in tube B containing synaptosomes maintained in external solution (37 °C, 45 min); no differences in background fluorescence were found between tubes B and C. After the second wash, pellets were resuspended in 400 µl of 2% PFA in PBS and maintained at 4 °C in the dark. Samples were acquired at Guava EasyCyte flow cytometer (EMD Millipore) and the cLTP analyzed using Incyte software (EMD Millipore) (Supplementary Fig. 7, Additional file 1).

### Statistical analyses

Statistical analyses were performed using GraphPad Prism version 9.1.0 software. *T* test two-tailed, one-way ANOVA with Dunnett’s multiple comparison test, or two-way ANOVA with Tukey’s multiple comparison test were used to detect significant differences between groups. Data were then expressed as the mean ± SD, and for all statistical analyses *p* = 0.05 was considered as statistically significant.

## Results

### AβO and TauO binding to human synaptosomes

**Fig. 1:**
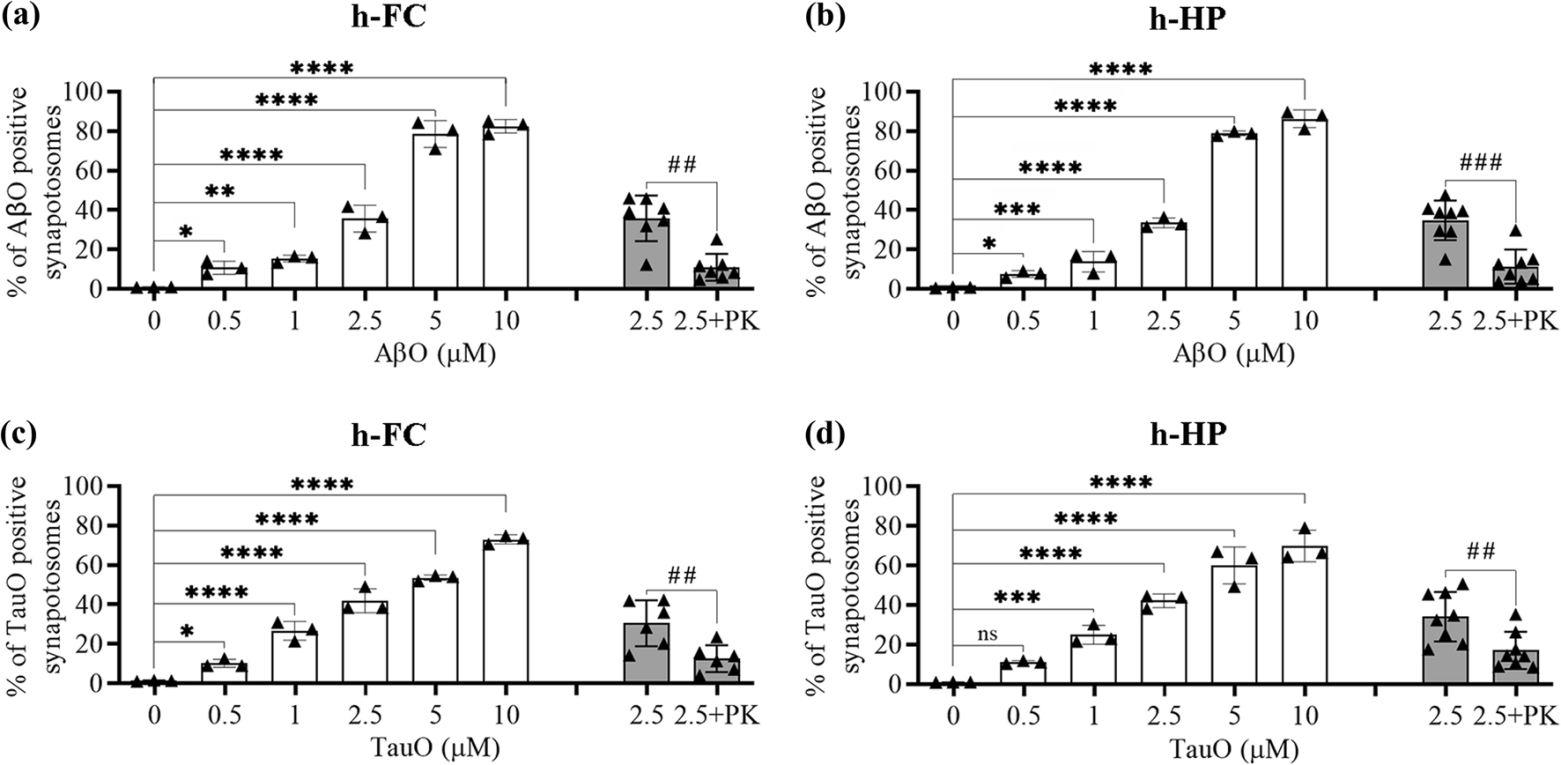
Characterization of dose dependent AβO and TauO binding to human synapses. **a** White columns—AβO binding to a pool of synaptosomes isolated from human FC samples. Data represent the mean ± SD; *P = 0.05, **P = 0.0075, and ****P < 0.0001 compared with synaptosomes not challenged with AβO (biological replicates *n* = 5; independent experiments *n* = 3; one-way ANOVA plus Dunnett’s multiple comparisons); gray columns—proteinase K (PK) enzymatic digestion of human FC synaptosomes post-challenge with AβO 2.5 µM. Data represent the mean ± SD; **P = 0.0023 compared with control (biological replicates *n* = 5; independent experiments *n* = 7; paired *t*-test, two-tailed). **b** White columns—AβO binding to a pool of synaptosomes isolated from human HP samples. Data represent the mean ± SD; *P = 0.05, ***P = 0.0010, and ****P < 0.0001 compared with synaptosomes not challenged with AβO (biological replicates *n* = 6; independent experiments *n* = 3; one-way ANOVA plus Dunnett’s multiple comparisons); gray columns—proteinase K (PK) enzymatic digestion of human HP synaptosomes post-challenge with AβO 2.5 µM. Data represent the mean ± SD; ***P = 0.0002 compared with control (biological replicates *n* = 6; independent experiments *n* = 8; paired *t*-test, two-tailed). **c** White columns—TauO binding to a pool of synaptosomes isolated from human FC samples. Data represent the mean ± SD; *P = 0.0329, and ****P < 0.0001 compared with synaptosomes not challenged with TauO (biological replicates *n* = 5; independent experiments *n* = 3; one-way ANOVA plus Dunnett’s multiple comparisons); gray columns—proteinase K (PK) enzymatic digestion of human FC synaptosomes post-challenge with TauO 2.5 µM. Data represent the mean ± SD; **P = 0.0086 compared with control (biological replicates *n* = 5; independent experiments *n* = 6; paired *t*-test, two-tailed); **d** White columns—TauO binding to a pool of synaptosomes isolated from human HP samples. Data represent the mean ± SD; ***P = 0.0009, and ****P < 0.0001 compared with synaptosomes not challenged with TauO (biological replicates *n* = 6; independent experiments *n* = 3; one-way ANOVA plus Dunnett’s multiple comparisons); gray columns—proteinase K (PK) enzymatic digestion of human HP synaptosomes post-challenge with TauO 2.5 µM. Data represent the mean ± SD; **P = 0.0081 compared with control (biological replicates *n* = 6; independent experiments *n* = 8; paired *t*-test, two-tailed)

To evaluate synaptic sensitivity to AβO and TauO, we used flow cytometry to assess their binding to synaptosomes isolated from frozen human FC and HP. Synaptosomes were incubated with increasing concentrations of oligomers (0.5–1–2.5–5–10 µM). As shown in Fig. 1 and supplementary Fig. 1 (Additional file 1), we observed dose-dependent increases in the percentages of binding of AβO (Fig. 1a, b white columns; Fig. Sup1a, b) and TauO (Fig. 1c, d white columns; Fig. Sup1c, d) to human FC and HP synaptosomes. Similar results were obtained when we incubated synaptosomes from mouse FC and HP with AβO and TauO (Supplementary Fig. 2, Additional file 1). To evaluate whether AβO and TauO were internalized or remained at the surface, we subjected the human synaptosomes to PK digestion. As shown in Fig. 1, both AβO (Fig. 1a, b gray columns) and TauO (Fig. 1c, d, gray columns) were accessible to enzymatic digestion in synaptosomes from human FC and HP, suggesting that both oligomeric species remained mostly at the external surface of the synaptosome, at least for the 60 min time frame of our experimental procedure. These data indicate that the methodology used in the present work allowed us to characterize dose dependent AβO and TauO binding at the surface of isolated human or murine synaptosomes.

### AβO and TauO synaptic binding dynamics in isolated synaptosome, primary neurons and in vivo models

#### A: isolated synaptosomes

**Fig. 2:**
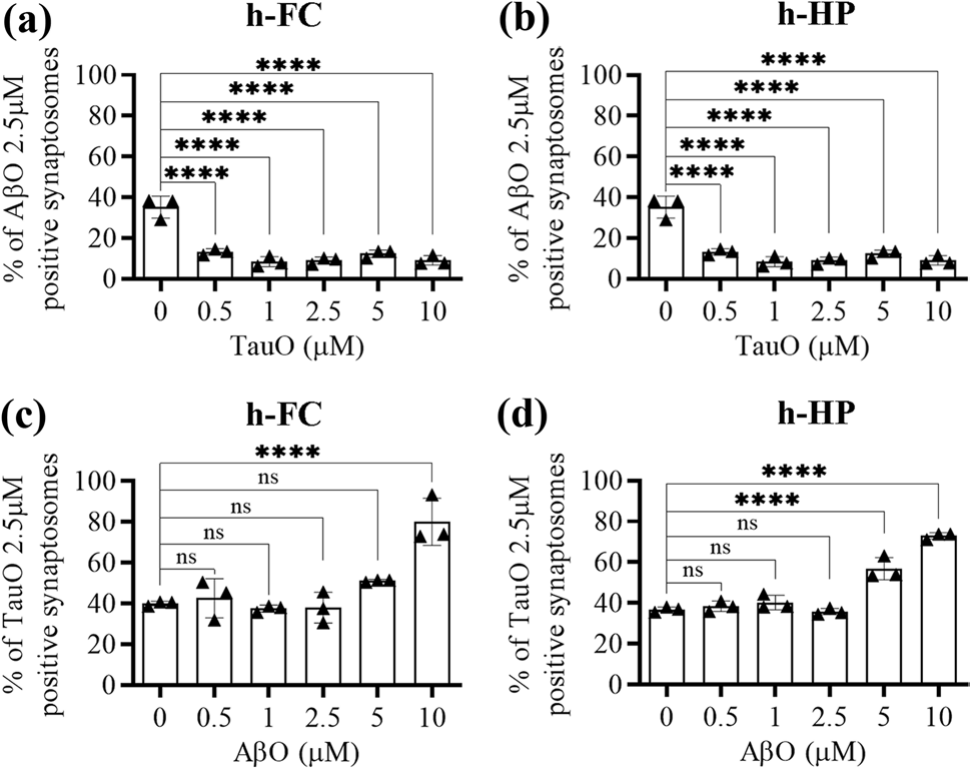
TauO reduces AβO binding to human FC and HP synaptosomes, while AβO increases TauO binding. Effect of increasing TauO concentrations (0.5–1–2.5–5–10 µM) on AβO binding to synaptosomes isolated from human **a** FC and **b** HP samples. Effect of increasing AβO concentrations (0.5–1–2.5–5–10 µM) on TauO binding to synaptosomes isolated from human **c** FC and **d** HP samples. Data represent the mean ± SD; ***P = 0.002, and ****P < 0.0001 compared with synaptosomes not challenged with AβO and TauO (biological replicates *n* = 5–6; independent experiments *n* = 3; ordinary one-way ANOVA plus Dunnett’s multiple comparison test)

**Fig. 3:**
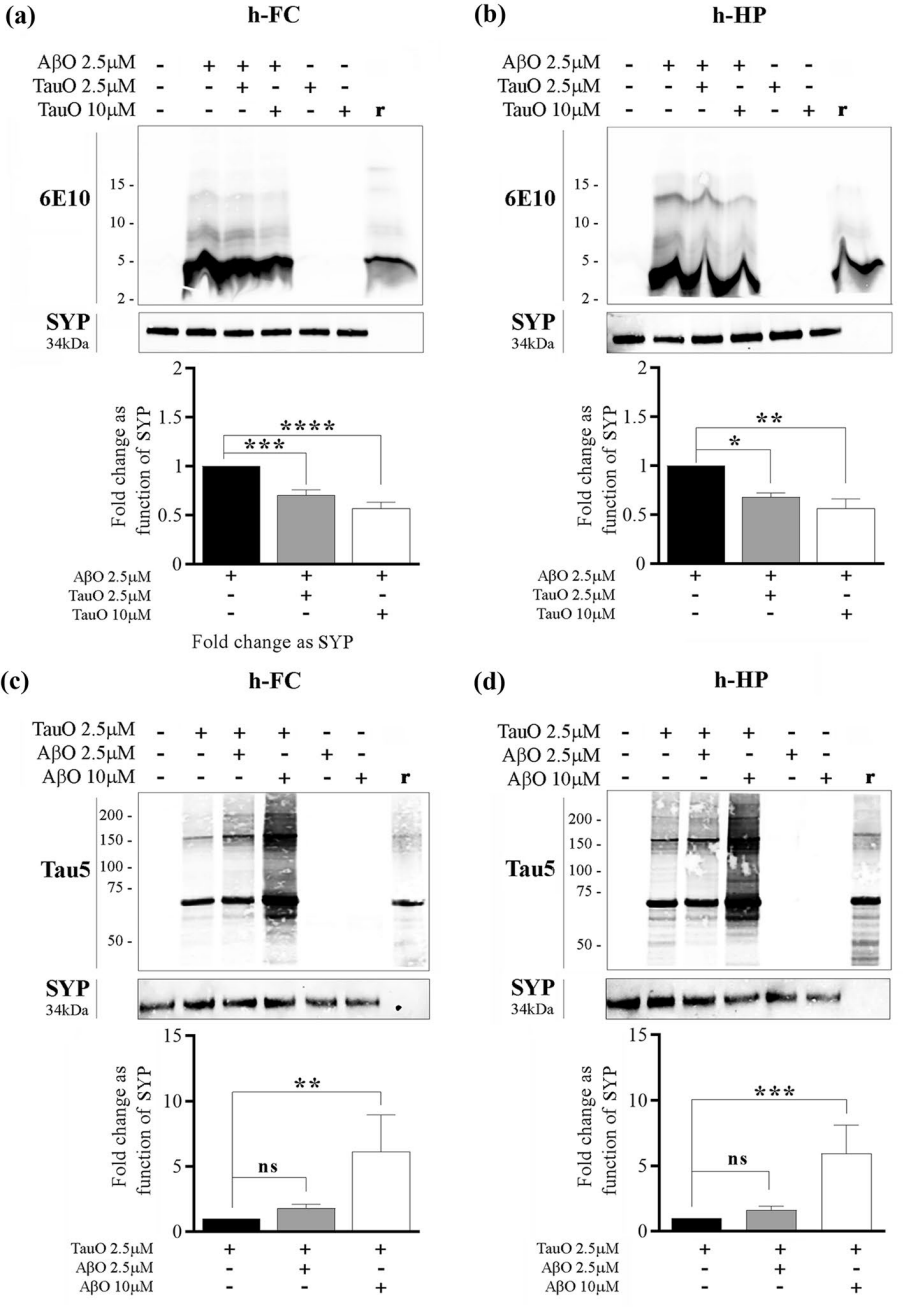
Western blot analyses confirming changes in AβO/TauO synaptic binding in the presence of each other*.* **a**, **b** Representative western blots (upper panels) and relative densitometric analyses (lower panels) of oligomeric species (selected area of quantification from about 8 kDa to about 16 kDa) to evaluate the percentages of AβO 2.5 µM bound to synaptosomes isolated from human **a** FC and **b** HP in the presence of TauO 2.5 and 10 µM. Data represent the mean ± SD; *P = 0.0109, **P = 0.0015, ***P = 0.0002, and ****P < 0.0001 compared with control synaptosomes (challenged with AβO alone) (biological replicates *n* = 5–6; independent experiments *n* = 5; one-way ANOVA plus Dunnett’s multiple comparison test); **c**, **d** Representative western blots (upper panels) and relative densitometric analyses (lower panels) of oligomeric species (selected area of quantification from about 60 kDa to up) to evaluate the percentages of TauO 2.5 µM bound to synaptosomes isolated from human **c** FC and **d** HP in the presence of AβO 2.5 and 10 µM. Data represent the mean ± SD; **P = 0.0034 (**c**), **P = 0.0007 (**d**), compared with control synaptosomes (challenged with TauO alone) (biological replicates *n* = 5–6; independent experiments *n* = 5; one-way ANOVA plus Dunnett’s multiple comparison test)

**Fig. 4:**
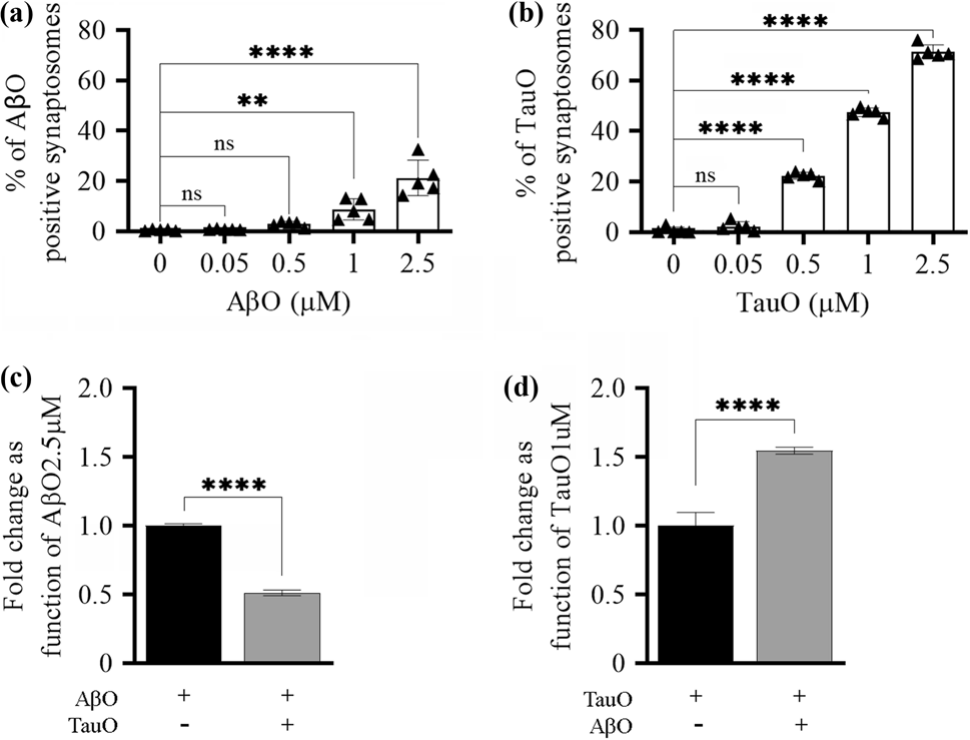
AβO/TauO synaptic binding in mice brain slices treated ex vivo with AβO plus TauO. Mice brain slices were treated with increasing concentrations of AβO and TauO, then synaptosomes were isolated and subjected to flow cytometric analysis to evaluate the dose-dependent binding percentages of **a** AβO and **b** TauO; Data represent the mean ± SD; **P = 0.0077 and ****P < 0.0001 compared with synaptosomes derived from untreated mouse brain slices (biological replicates *n* = 14–30; independent experiments *n* = 5; ordinary one-way ANOVA plus Dunnett’s multiple comparisons); mice brain slices were treated with a combination of AβO 2.5 µM and TauO 1 µM before the synaptosomes were isolated for flow cytometric analyses to evaluate the binding percentage of **c** AβO and **d** TauO. Data represent the mean ± SD; ****P < 0.0001 compared with brain slices challenged with AβO or TauO alone (biological replicates *n* = 10; independent experiments *n* = 2; paired *t*-test, two-tailed)

Synaptosomes isolated from human FC and HP were incubated with either AβO or TauO (both 2.5 µM) in the presence of increasing concentrations (0.5–10 µM) of TauO or AβO, respectively, and levels of the resulting binding was detected by flow cytometry. The percentage of AβO bound to human FC synaptosomes significantly decreased in the presence of all TauO concentrations (0.5–10 µM, Fig. 2a). Similar results were observed when AβO binding to human HP synaptosomes (Fig. 2b) was evaluated in the presence of increasing TauO concentrations (0.5–10 µM). On the other hand, TauO binding to synaptosomes isolated from human FC (Fig. 2c) and HP (Fig. 2d) was not reduced by the concomitant presence of low AβO levels (0.5–1–2.5 µM). Notably, higher AβO concentrations (5 and 10 µM) induced a trend of increase in TauO binding to FC (significant for AβO 10 µM) (Fig. 2b) and HP (significant for both AβO 5 and 10 µM) (Fig. 2d) synaptosomes. Similar results were observed in mouse synaptosomes (Supplementary Fig. 3, Additional file 1). To confirm these observations, we performed western blotting analyses on human synaptosomes post-challenged with either AβO or TauO (2.5 µM) in the presence of increasing concentrations (2.5 and 10 µM) of TauO and AβO, respectively. Our results showed that the amounts of AβO bound to human FC (Fig. 3a) and HP (Fig. 3b) synaptosomes decreased by 0.3- and 0.5-fold in the presence of TauO at 2.5 µM and 10 µM, respectively. Conversely, the amount of TauO bound to human FC (Fig. 3c) and HP (Fig. 3d) synaptosomes was not significantly affected by AβO 2.5 µM but increased by ~ five fold in the presence of 10 µM AβO. Moreover, human FC and HP synaptosomes incubated with AβO 2.5 µM in the presence of increasing concentrations (0.5–5 µM) of α-synuclein oligomers (α-synO) failed to show any reduction trend in synaptosomes positive for AβO, suggesting that the decrease in synaptic AβO binding is relatively specific to TauO (Supplementary Fig. 4a, b, Additional file 1). Furthermore, Human FC and HP synaptosomes incubated with TauO 2.5 µM in the presence of increasing concentrations (0.5–5 µM) of α-synO failed to show any increase trend in synaptosomes positive for TauO, suggesting that the increase in TauO binding is relatively specific to AβO (Supplementary Fig. 4c, d, Additional file 1).To determine if this phenomenon also occurred in a system in which the cytoarchitecture of the CNS is preserved, we studied the binding dynamics of AβO and TauO in brain slices prepared from 4-month-old C57BL/6J mice (*n* = 14). We treated brain slices for 1 h with 0.05–0.5–1–2.5 µM of labeled AβO or TauO before preparing synaptosomes for flow cytometry to assess binding. We observed dose-dependent increases in the percentages of AβO (Fig. 4a) and TauO (Fig. 4b) binding. We also treated mouse brain slices from 10 mice with a combination of AβO (2.5 µM) and TauO (1 µM) and observed that the amount of AβO bound to synaptosomes decreased by ~ 0.5 fold in the presence of TauO 1 µM (Fig. 4c). Consistent with the data described above, the amount of synaptosome-bound TauO increased by ~ 0.5-fold in the presence of AβO 2.5 µM (Fig. 4d).

#### B: mouse cortical primary neurons

**Fig. 5:**
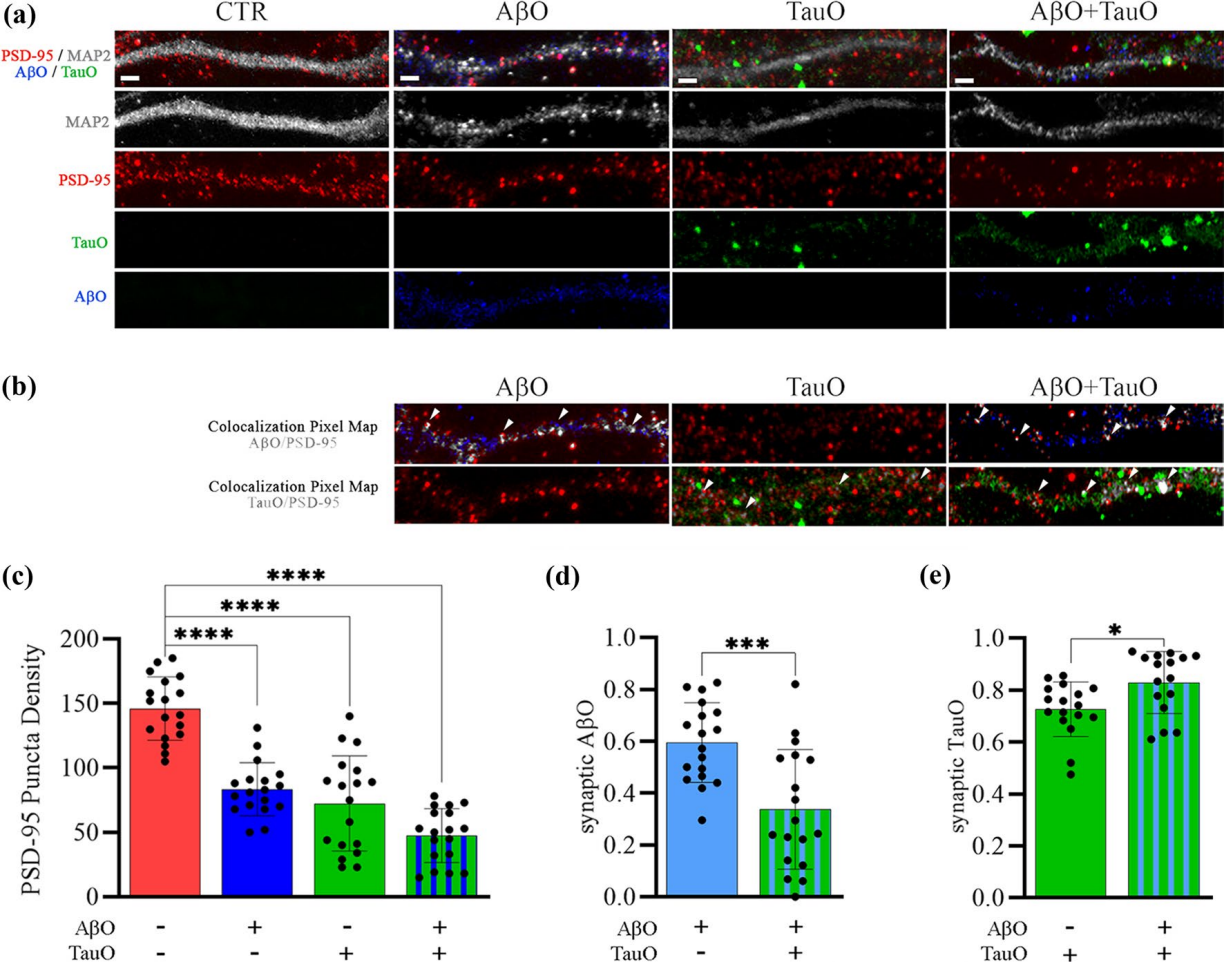
Immunofluorescence analyses of AβO and TauO binding dynamics in mouse primary neuron synapses. **a** Representative confocal images of neuronal projections (30-µm long) from mouse primary cortical neurons treated with AβO 2.5 µM (blue), TauO 2.5 µM (green) and their combination (blue + green) and stained for MAP2 (gray) and PSD95 (red); original magnification 63 × , scale bar 2 µm. **b** Representative pixel maps of AβO/PSD95 and TauO/PSD95 showing colocalizations in gray (white arrowheads) of neuronal projections showed in a . **c** Quantitative analysis of PSD95 puncta within 30-µm length of neuronal projections randomly selected from mouse primary cortical neurons treated with AβO 2.5 µM (blue), TauO 2.5 µM (green), and their combination (blue + green) as compared with untreated cells (red). Data represent the mean ± SD, ****P < 0.0001 compared with control cells (neuronal projections analyzed *n* = 18; independent experiments *n* = 3; ordinary one-way ANOVA plus Dunnett’s multiple comparisons). **d** Quantitative analysis of synaptic AβO within 30-µm lengths of neuronal projections randomly selected from mouse primary cortical neurons treated with AβO 2.5 µM (blue) and the combination of AβO 2.5 µM and TauO 2.5 µM (blue + green); data represent the mean ± SD, ***P = 0.0004 (neuronal projections analyzed *n* = 18; independent experiments *n* = 3; unpaired *t*-test, two-tailed). **e** Quantitative analysis of synaptic TauO within 30-µm lengths of neuronal projections randomly selected from mouse primary cortical neurons treated with TauO 2.5 µM (green) and the combination of AβO 2.5 µM and TauO 2.5 µM (blue + green); Data represent the mean ± SD, *P = 0.0128 (neuronal projections analyzed *n* = 18; independent experiments *n* = 3; unpaired *t*-test, two-tailed)

We performed immunofluorescence analyses of mouse primary cortical neurons after labeling the neurites and synaptic spines with MAP2 and PSD95 antibodies, respectively. Confocal images of neurons treated with pre-labeled AβO and TauO (both 2.5 µM) showed neuronal degeneration as compared with untreated cultures. MAP2 staining pointed out the neuronal atrophy with a reduction of neuronal projections after AβO and/or TauO. Moreover, PSD95 in neurons treated with oligomers showed a loss of synaptic distribution which was exacerbated by the co-incubation of AβO and TauO (Supplementary Fig. 5, Additional file 1). This effect was exacerbated when neurons were simultaneously incubated with both oligomeric species. Figure 5 shows representative high-magnification neurite images following treatment with pre-labeled AβO and/or TauO (both 2.5 µM) (Fig. 5a) and the extent of colocalization of the oligomers with PSD95 (Fig. 5b). Co-treatment with AβO and TauO led to decreased PSD95 labeling as compared with untreated cultures. Again, this phenomenon was more evident when neurons were treated with both oligomer species, which was confirmed by quantitative analysis (Fig. 5c). We quantified the synaptic AβO and TauO in all our experimental conditions. When we compared neurons treated with AβO alone and with AβO plus TauO, we observed decreases in the synaptic AβO from 0.56 to 0.36 (Fig. 5d). On the other hand, the presence of AβO resulted in an increase in synaptic TauO from 0.72 to 0.87 (Fig. 5e).

#### C: 3xTgAD mice

**Fig. 6:**
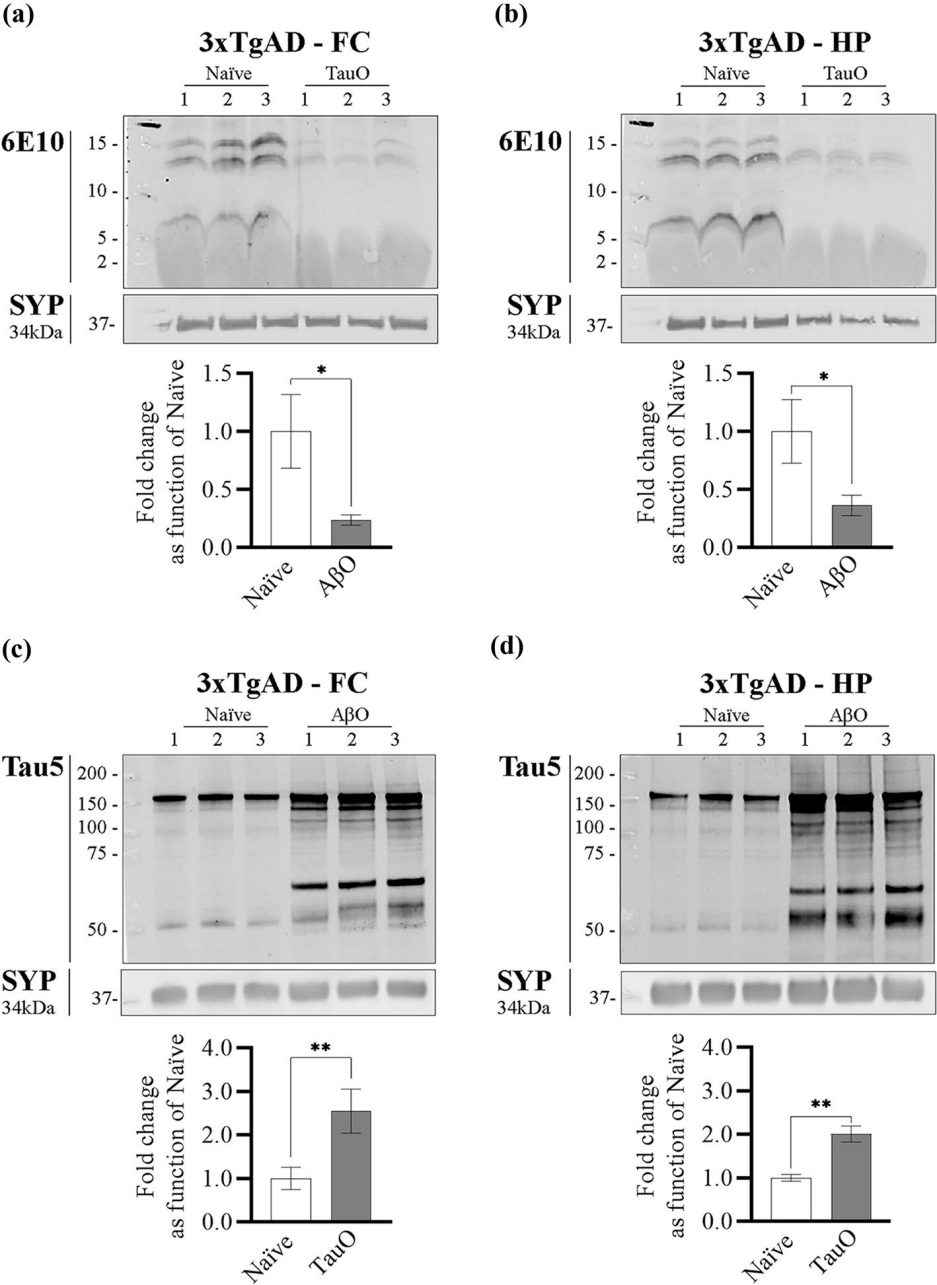
In vivo treatment of 3xTgAD mice with AβO and TauO. **a**, **b** Representative western blots (upper panels) and relative densitometric analyses (lower panels) of oligomeric species (selected area of quantification from about 8 kDa to about 16 kDa) to evaluate the levels of AβO bound to synaptosomes from **a** FC and **b** HP of 3xTgAD mice ICV with TauO 0.55 µM; data represent the mean ± SD; *P = 0.0147 (**a**) and *P = 0.0186 (**b**) as compared with FC and HP synaptosomes isolated from 3xTgAD naïve mice (biological replicates *n* = 3/group; independent experiments *n* = 3; unpaired *t*-test, two-tailed); **c**, **d** Representative western blots (upper panels) and relative densitometric analyses (lower panels) of oligomeric species (selected area of quantification from about 60 kDa to up) to evaluate the levels of TauO bound to synaptosomes from **c** FC and **d** HP of 3xTgAD mice ICV with AβO 0.55 µM. Data represent the mean ± SD; **P = 0.009 (**c**) and **P = 0.001 as compared with FC and HP synaptosomes isolated from 3xTgAD naïve mice (biological replicates *n* = 3/group; independent experiments *n* = 7; unpaired *t* test, two-tailed)

To evaluate AβO and TauO binding dynamics in vivo we injected ICV 16-month-old 3xTgAD mice with 3 µl of 0.55 µM of either AβO or TauO and performed western blot analyses on the synaptosomal protein extracts as previously described [29]. Naïve 3xTgAD mice were used as control. We collected the brain regions 24-h post-ICV injections and extracted the synaptosomal proteins from hippocampus and frontal cortex. Western blot analyses performed on synaptosomal proteins extracted from 3xTgAD mice ICV injected with TauO showed a decrease in AβO levels in both frontal cortex (Fig. 6a) and hippocampus (Fig. 6b) synaptosomes of ~ 0.8 and ~ 0.65 fold, respectively, as compared with 3xTgAD naïve mice. On the other hand, western blot analyses performed on synaptosomal protein extracted from 3xTgAD mice injected ICV with AβO showed an increase in TauO levels in both frontal cortex (Fig. 6c) and hippocampus (Fig. 6d) synaptosomes of ~ 2.5 and ~ two fold, respectively, as compare with 3xTgAD naïve mice.

Collectively, these results show differential synaptic binding dynamics for AβO and TauO in three distinct model systems (isolated synaptosomes, in vitro primary neurons and in vivo), where TauO negatively affected the binding of AβO to synaptosomes, whereas high concentrations of AβO promoted the synaptic binding of TauO.

### Effect of PK pre-treatment on AβO and TauO synaptic binding

**Fig. 7:**
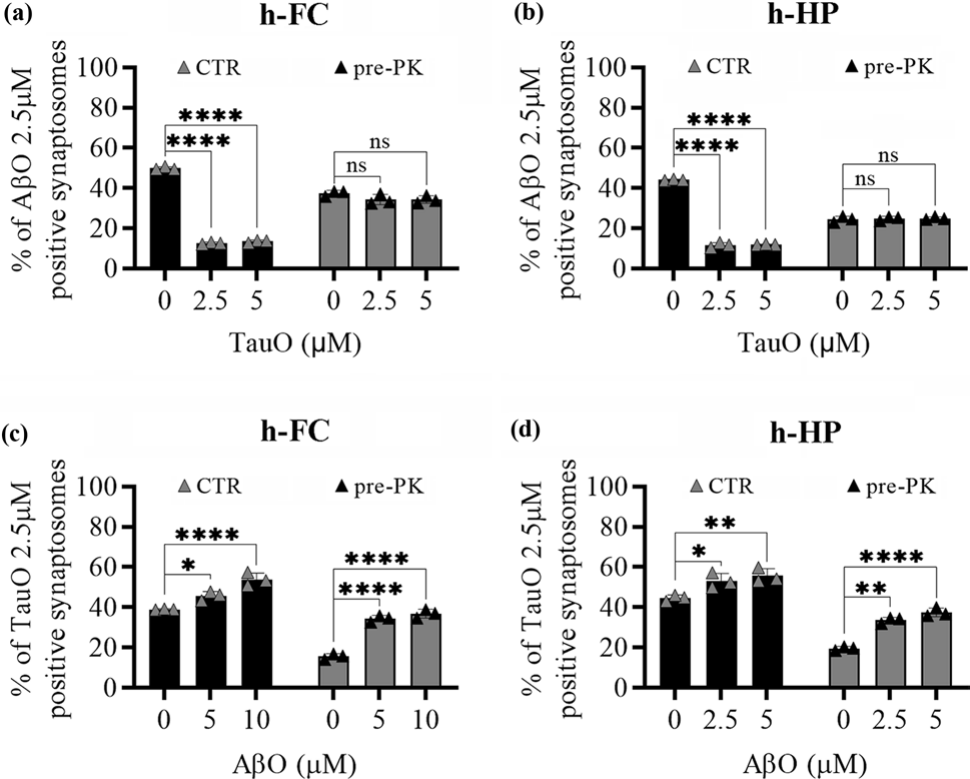
Effects of PK pret-reatment of human FC and HP synaptosomes on AβO/TauO binding dynamics. The surface protein component of human synaptosomes was digested with 1 mg/ml PK before the synaptosomes were challenged with AβO and/or TauO, and the resulting binding was detected by flow cytometric analysis. CTR, synaptosomes not PK pre-treated, pre-PK, synaptosomes PK pre-treated. Binding dynamics of AβO 2.5 µM to human **a** FC and **b** HP synaptosomes in the presence of increasing concentrations of TauO (2.5 and 5 µM); Data represent the mean ± SD; ****P < 0.0001 compared with control synaptosomes (challenged with AβO and TauO alone) (biological replicates *n* = 5–6; independent experiments *n* = 3; two-way ANOVA plus Tukey’s multiple comparison test). Binding dynamics of TauO 2.5 µM to human **c** FC and **d** HP synaptosomes in the presence of increasing concentrations of AβO (5 and 10 µM). Data represent the mean ± SD; *P = 0.05, **P = 0.0083 (**d**—CTR samples) and **P = 0.0015 (**d**—PK samples), and ****P < 0.0001 compared with control synaptosomes (challenged with AβO and TauO alone) (biological replicates *n* = 5–6; independent experiments *n* = 3; two-way ANOVA plus Tukey’s multiple comparison test)

To understand if AβO and TauO binding occur on a protein substrate, we pre-treated human FC and HP synaptosomes with 1 mg/ml of PK for 30 min at 37 °C to remove cell-surface proteins. Then we challenged the digested synaptosomes with increasing concentrations of AβO and TauO (0.5–10 µM) and assessed their binding with flow cytometry. PK pre-treatment showed a trend in reducing AβO and TauO binding to human FC and HP synaptosomes, especially at the 2.5 µM and 5 µM concentrations of both oligomers (Supplementary Fig. 6, Additional file 1). Based on these observations, PK pre-digested synaptosomes were challenged with either AβO or TauO (both 2.5 µM) in the presence of increasing concentrations of TauO (0.5–10 µM) and AβO (2.5–5 µM) respectively. Figure 7 shows that AβO binding to human FC (Fig. 7a) and HP (Fig. 7b) synaptosomes devoid of surface proteins by the PK pre-treatment was not affected by the presence of TauO either at 2.5 µM or 5 µM. On the other hand, the binding of TauO to PK pre-treated human FC (Fig. 7c) and HP (Fig. 7d) synaptosomes was increased by the concomitant presence of AβO (5 µM and 10 µM), similarly with what we observed in synaptosomes not pre-treated with PK. Collectively these data suggest that TauO can outcompete AβO from a protein substrate, while the ability of high AβO levels to increase synaptic TauO recruitment is related to a non-protein substate.

### TauO-induced cLTP suppression is not affected by AβO

**Fig. 8:**
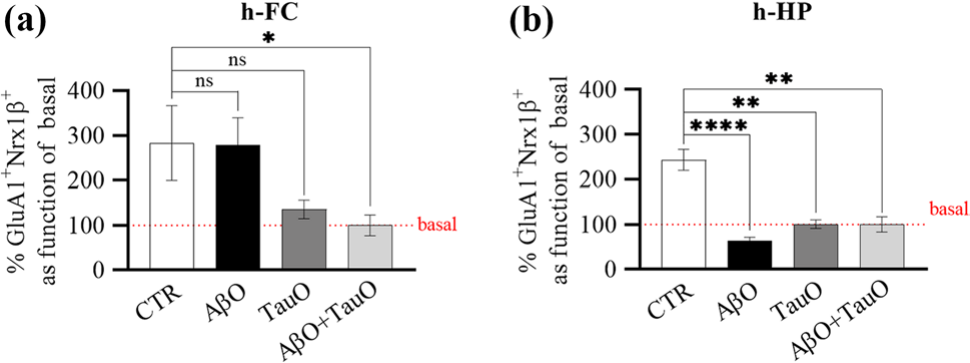
Impact of AβO and TauO on physiological synaptic transmission of human synapses. FASS-LTP identifies potentiated synapses by tracking GluA1 and Nrx1ß surface expression in size-gated synaptosomes. Percentages of GluA1^+^Nrx1ß^+^ size-gated synaptosomes isolated from human **a** FC and **b** HP post-challenged with AβO and/or TauO 0.5 µM. Data represent the mean ± SEM; *P = 0.05, **P = 0.0088 (**b** TauO vs CTR) and **P = 0.0065 (**b** AβO + TauO vs CTR) and ****P < 0.0001 compared with the basal levels (biological replicates *n* = 5–6; independent experiments *n* = 3; ordinary one-way ANOVA plus Dunnett’s multiple comparison test)

To assess the impact of the oligomers on synaptic function, we analyzed the effects of AβO and TauO on cLTP in stimulated synaptosomes with fluorescence analysis of single-synapse long-term potentiation (FASS-LTP) [42–44] (Fig. 8 and Supplementary Fig. 8). Human FC and HP synaptosomes were challenged with AβO and/or TauO (both 0.5 µM) prior to FASS-LTP analyses. AβO did not affect cLTP in human FC synaptosomes (Fig. 8a) but suppressed it in human HP synaptosomes (Fig. 8b). Notably, TauO treatment suppressed cLTP in HP synaptosomes and showed a trend of suppression in FC synaptosomes. Consistent with the binding results, these data show that TauO-induced cLTP suppression was not affected by the concomitant presence of AβO in human FC or HP synaptosomes.

## Discussion

This study investigated synaptic binding dynamics for AβO and TauO to determine the level of interaction between these oligomer species at the synapses, yielding seven main findings. (1) Experiments assessing dose-dependent AβO and TauO binding to human FC and HP synaptosomes demonstrated that binding was mostly limited to the synaptosome surface within the 1h time frame of our experimental procedure. (2) Flow cytometry and western blotting studies demonstrated that the amount of AβO bound to FC and HP synaptosomes significantly decreased in presence of TauO. Although lower levels of AβO did not alter TauO binding, higher AβO concentrations resulted in increased TauO binding to human synapses. (3) These findings were confirmed in mouse brain slices with intact neuronal architecture. (4) Immunofluorescence analyses of mouse primary cortical neurons treated with AβO and/or TauO fully supported these data. (5) Western blot analyses of synaptosomal proteins derived from hippocampus and frontal cortex of 3xTgAD mice injected ICV with AβO and TauO reinforced these findings in vivo. (6) Pre-treatment with PK showed that the effect of TauO in reducing AβO synaptic binding was lost after the depletion of synaptosome surface proteins; on the other hand, the effect of AβO in increasing TauO synaptic binding was not affected. (7) Functional FASS-LTP studies revealed that TauO-induced cLTP suppression was not affected by the presence of AβO. Taken together, our results provide deeper insight into the interplay between AβO and TauO that contributes to synaptic dysfunction, which is the basis of the cognitive decline in AD [3, 5]. This study is focused on hippocampus and frontal cortex which are considered vulnerable brain regions to AD. We aim to study in the future less AD vulnerable brain regions, such as cerebellum and primary sensory cortex, to identify possible difference in AβO and TauO synaptic binding dynamics.

Synaptic dysfunction, neuronal death, and subsequent memory loss are likely due to cross talk between AβO and TauO [3, 7, 45, 46]. This underscores the importance of clarifying whether AβO and TauO act independently, in tandem, or synergistically. Some studies reported that AβO and TauO individually contribute to the characteristic AD impairment of synaptic plasticity and subsequent memory dysfunction [17, 18, 47, 48], while others showed that prior AβO accumulation is necessary for TauO-induced neuronal alterations [3, 8, 16, 19]. Moreover, one study supports the hypothesis that tau is an important mediator of AβO-induced neurodegeneration in AD [49]. An electrophysiological investigation by Fa et al. concluded that simultaneous suppression of hippocampal Schaffer collateral high-frequency stimulation LTP and memory impairment could be induced by low concentrations of AβO and TauO that do not normally perturb synaptic function, suggesting that the two species synergistically exert their effects [13]. A 2019 report found that the neuronal impact of TauO dominated over AβO in several in vivo models [50]. Here we provide the first evidence of differential binding dynamics for AβO and TauO at human synapses, where TauO seems to overcome AβO effects, while progressive increases in AβO reinforce the effects of TauO. This last phenomenon was demonstrated by increased amounts of TauO at human synapses promoted by AβO in a dose-dependent manner. These events were confirmed in mouse synapses derived from mouse brain slices, mouse brains post-ICV injection with either AβO or TauO, and cortical primary neurons. AβO are unique to AD and have not been described in other tauopathies [51]. Based on this and the findings described here, it is prudent to speculate that the differential temporal appearance/prevalence of AβO and TauO in AD may not be incidental. A recent investigation of how AβO uniquely seeds TauO assembly found that 100–200 nM of AβO facilitated TauO seeding [52]. Here we show that AβO increased the amount of TauO that bound to human synaptosomes. This phenomenon was also observed in PK pre-treated synaptosomes suggesting that in the absence of surface proteins AβO was still able to recruit TauO at the human synapses. Given the reported trans-synaptic spreading of TauO [53, 54], this effect of AβO on TauO binding dynamics may explain the progressive prevalence of TauO during later stages of AD.

Several cell-surface proteins have been identified as binding sites for AβO and TauO [55, 56]. Among these is the low-density lipoprotein receptor-related protein (LRP1), which is highly expressed in the postsynapses and reportedly plays a central role in AD pathogenesis. Two compelling studies described LRP1’s involvement in key AD pathologic events including Aβ clearance, amyloid precursor protein metabolism, matrix metalloproteinase 9 function, the pathogenic role of apolipoprotein E, modulation of glutamate receptor function, calcium homeostasis, and cellular TauO uptake and trans-synaptic spread [55, 57]. The present results demonstrated that sufficient levels of TauO outcompete AβO when the latter is bound to a synaptic protein substrate, while AβO independently of their binding to the synaptosome surface proteins exacerbate TauO synaptic binding. These findings suggest that synaptic membrane proteins mediate AβO and TauO binding dynamics, although further studies are necessary to identify specific targets at the synaptic membrane.

Learning and memory depend on synaptic strengthening in response to activity, but these critical processes are impaired when synapses are dysfunctional due to the actions of AβO and TauO, as in AD. Investigating human synaptic transmission is difficult given the evident methodological limitations. Here, we analyzed cLTP in synaptosomes isolated from human samples using FASS-LTP, a novel approach proposed by the Cotman group [42, 43] and optimized by the Krishnan group [44]. Conventional long-term potentiation (LTP) is a current electrophysiological approach widely used for studying molecular mechanisms of hippocampal synaptic plasticity in intact neural circuitries [43]. These characteristics make the LTP a more comprehensive methods which take in account electrical properties of neurons, while the cLTP measured with FASS-LTP is specifically limited to the synaptic interaction GluA1–Nrx1β. Our results show that TauO induced cLTP suppression, regardless of the effect of AβO on synaptic function. Specifically, TauO showed a trend of cLTP suppression in human synaptosomes isolated from FC that was exacerbated when in presence of AβO which alone showed no effect on the cLTP. These data suggest that TauO may have more severe effects on GluA1–Nrx1β interaction in human FC synaptosomes compared with AβO. Moreover, the suppressive effect of TauO on cLTP was exacerbated in human HP synaptosomes, where AβO also significantly suppresses cLTP. These differences between FC and HP could be related to a different sensitivity of the receptors, based on levels [58]. We recently reported that the absence of TauO is at the basis of the synaptic functional integrity and cognitive competency in a unique group of individuals who maintain cognitive ability despite the presence of all the neuropathological features of AD [44]. The data presented here, further demonstrate that in advanced stages of AD, when TauO levels progressively increase and reach a critical mass, they become the most toxic species driving synaptic dysfunction and clinical manifestations. Perhaps this could explain the disappointing results of Aβ-directed therapeutics in patients with late-stage AD when cognitive decline is already manifest [2].

## Conclusions

In conclusion, these findings suggest a link between the temporal occurrence of AβO and TauO and the progression of AD, with direct implications for how synaptic disruption driven by the binding of these oligomers underlies dementia pathogenesis in a differential and yet interactive way. Our work highlights the importance of developing therapeutic approaches that target TauO; such treatments could be beneficial for both late-stage AD and other tauopathies.

## Supplementary Information

Below is the link to the electronic supplementary material.

Supplementary file1 (DOCX 25 KB)

Supplementary file2 (TIF 9288 KB)

Supplementary file3 (TIF 10064 KB)

Supplementary file4 (TIF 4552 KB)

Supplementary file5 (TIF 4628 KB)

Supplementary file6 (TIF 8280 KB)

Supplementary file7 (TIF 5390 KB)

Supplementary file8 (TIF 5041 KB)

Supplementary file9 (TIF 5515 KB)

Supplementary file10 (TIF 5348 KB)

## Data Availability

All data generated or analyzed during this study are included in this publication and/or are available from the corresponding author on reasonable request.

## Abbreviations

aCSF: Artificial cerebrospinal fluid
ACTB: Actin
AD: Alzheimer’s disease
AβO: Amyloid β oligomers
BSA: Bovine serum albumin
AMPARs: α-Amino-3-hydroxy-5-methyl-4-isoxazoleropionic acid receptors
CERAD: Consortium to establish a registry of AD
FASS-LTP: Fluorescence analysis of single-synapse long-term potentiation
FC: Frontal cortex
HBK: HEPES buffered Krebs-like
HFP: 1,1,1,3,3,3-Hexafluoro-2-propanol
NMDG: *N*-Methyl-d-glucamine
HP: Hippocampus
ICV: Intracerebroventricular
MAP2: Microtubule-associated protein 2
NMDAR: *N*-Methyl d-aspartate receptor
Nrx1β: Neurexin-1β
PBS: Phosphate-buffered saline
PBST: Phosphate-buffered saline with tween
PFA: Paraformaldehyde
PK: Proteinase K
PSD95: Postsynaptic density
RIPA: Radioimmunoprecipitation assay buffer
SYPH: Synaptophysin
TauO: Tau oligomers
TBST: Tris-buffered saline solution with tween
