# Supplementary material for: Aβ/tau oligomer interplay at human synapses supports shifting therapeutic targets for Alzheimer’s disease
Source: Cell Mol Life Sci. 2022 Apr 4;79(4):222. doi: 10.1007/s00018-022-04255-9 (PMC8979934; doi:10.1007/s00018-022-04255-9)
Supplement: Supplementary file 1 — Supplementary file1 (DOCX 25 KB) [file 18_2022_4255_MOESM1_ESM.docx]

**Additional file 1**

**Aβ/tau oligomer interplay at human synapses supports shifting therapeutic targets for Alzheimer’s disease**

Michela Marcatti, Anna Fracassi, Mauro Montalbano, Chandramouli Natarajan, Balaji Krishnan, Rakez Kayed, and Giulio Taglialatela*

***To whom correspondence should be addressed:** [gtaglial@utmb.edu](mailto:gtaglial@utmb.edu)

**Supplementary Contents**

**Supplementary Table 1.** Clinical information on the human brain samples used in this study

**Supplementary Methods**.

**Supplementary Methods** 1. Quality control of synaptosomes isolated from human brain samples by electron microscopy, flow cytometry, and western blot.

**Supplementary Methods 2.** Characterization of AβO and TauO binding to human synaptosomes isolated from single human subjects.

**Supplementary Figures**

**Supplementary Fig. 1.** Flow cytometric characterization of AβO and TauO binding to human synapses.

**Supplementary Fig. 2.** Characterization of AβO and TauO binding to mouse synaptosomes

**Supplementary Fig. 3.** α-synO failed to show any reduction in the binding of AβO to human HP synaptosomes.

**Supplementary Fig. 4.** TauO reduce AβO binding to mouse frontal cortex (FC) and hippocampus (HP) synaptosomes, while AβO increase TauO synaptic binding

**Supplementary Fig. 5.** Confocal microscopy of mouse primary cortical neurons

**Supplementary Fig. 6.** The effects of PK pretreatment of human frontal cortex (FC) and hippocampus (HP) synaptosomes on AβO and TauO binding

**Supplementary Table 1.** Clinical information on the human brain samples used in this study

| Case # | Diagnosis | Age | | Sex | | Braak  stage | | MMSE  score | PMI (h) | | FC | HP |
| --- | --- | --- | --- | --- | --- | --- | --- | --- | --- | --- | --- | --- |
| 2467 | ctrl | 99 | | F | | 3 | | 28 | 4.5 | | yes | yes |
| 2553 | ctrl | 100 | | M | | 2 | | 28 | 4 | | no | yes |
| 2682 | ctrl | 90 | | F | | 2 | | 29 | 9 | | yes | yes |
| 2755 | ctrl | 95 | | F | | 2 | | 29 | 18 | | yes | yes |
| 2953 | ctrl | 100 | | F | | 3 | | 27 | 2.5 | | yes | yes |
| 3200 | ctrl | 90 | | M | | 2 | | 20 | 4.5 | | yes | yes |
|  |  |  |  | |  | |  |  |  |  |  |  |

Braak stage: a measure of the number and location of tau tangles and Aβ plaques in the brain (range: 1–6); MMSE: Mini-Mental State Examination (administered within the last year, range 0–30). PMI: postmortem interval (mean 7.08 h).

**Supplementary Methods 1. Quality control of synaptosomes isolated from human brain samples by electron microscopy, flow cytometry, and western blot.**

Ultrastructure analysis of synaptosomes was performed with 5 μl drops adsorbed on a 200-mesh coated resin grid (FCF 200 – CU Formvar/Carbon, Electron Microscopy Sciences, Hatfield, PA, USA) for 10 min at room temperature (RT). Grids were blotted with filter paper and stained with 2% aqueous uranyl acetate (cat# 541-09-3, Electron Microscopy Sciences) for negative staining for 1 min at RT. The uranyl acetate was then removed using filter paper, and the grids were dried with warm regular light for 2 min. Images were acquired with a Philips CM-100 transmission electron microscope at 60 kV with an Orius SC2001 digital camera (Gatan, Pleasanton, CA, USA). a) Representative transmission electron micrographs of presynapses (yellow), synaptic vesicles (SV), postsynaptic density (PSD) (red line) and the postsynapses (green) of synaptosomes isolated from the human frontal cortex (left), and hippocampus (right).

Synaptosomes were acquired by a Guava EasyCyte 8 flow cytometer (EMD Millipore, Burlington, MA) that contains a Class IIIb laser operating at 488 nm in continuous wave (CW) mode and a laser operating at 640 nm in CW mode. The relative size and granularity of synaptosomes were determined by forward scatter (FSC) and side scatter (SSC) properties. FSC–SSC plots were generated and used to select particles matching the size of synaptosomes (0.75–5.0 µm) using calibrated beads (Spherotech Inc., Lake Forest, IL, USA). FSC, SSC, and fluorescent (Green [525 ± 30 nm] and Red2 [661 ± 19 nm]) signals were collected using log amplification. Identical FSC settings were used to acquire data on bead standards and samples. Small fragments and debris were excluded by establishing an FSC-H threshold (gain = 2). Settings for fluorescence amplification on Green and Red2 photomultiplier tube detectors were based on the emissions detected on size-based gated particles. Alexa-488 and Alexa-647 fluorochromes were detected by the Green and Red2 detectors, respectively. A total of 5,000 size-gated particles were collected for each sample, and analysis was performed using Incyte software (EMD Millipore). b) Representative flow cytometry plots showing the gating applied using standard size beads (2–3.3–5.11–7.56 µm) to collect synaptosomes between 1 and 5 μm; representative flow cytometry plots showing the distributions of synaptosomes isolated from human FC and human HP samples according to the gating protocol.

Synaptosomes were lysed with RIPA buffer and processed for western blot analyses. c) Representative western blot of PSD95, SYPH ACTB in proteins from total homogenate (left) and synaptosomes (right) of human FC (upper panel) and HP (lower panel).

**Supplementary Methods 2. Characterization of AβO and TauO binding to human synaptosomes isolated from single human subjects.**

a) Binding of AβO to synaptosomes isolated from FC of 5 human subjects; data represent the mean ± SD; *P = 0.038, ***P = 0.003, and ****P < 0.0001 compared with synaptosomes not challenged with oligomers (biological replicates n = 5; one-way ANOVA plus Dunnett’s multiple comparison test). b) Binding of AβO to synaptosomes isolated from HP of 6 human subjects; data represent the mean ± SD; **P = 0.019, and ****P < 0.0001 compared with synaptosomes not challenged with oligomers (biological replicates n = 6; one-way ANOVA plus Dunnett’s multiple comparisons). c) Binding of TauO to synaptosomes isolated from FC of 5 human subjects; data represent the mean ± SD; ***P = 0.0007, and ****P < 0.0001 compared with synaptosomes not challenged with oligomers (biological replicates n = 5; one-way ANOVA plus Dunnett’s multiple comparisons). d) Binding of TauO to synaptosomes isolated from HP of 6 human subjects; data represent the mean ± SD; *P = 0.0113, and ****P < 0.0001 compared with synaptosomes not challenged with oligomers (biological replicates n = 6; one-way ANOVA plus Dunnett’s multiple comparisons). e) Effect of increasing TauO concentrations (0.5–1–2.5–5–10 µM) on AβO binding to synaptosomes isolated from FC of 5 human subjects; data represent the mean ± SD; ****P < 0.0001 compared with synaptosomes not challenged with oligomers (biological replicates n = 5; one-way ANOVA plus Dunnett’s multiple comparisons). f) Effect of increasing TauO concentrations (0.5–1–2.5–5–10 µM) on AβO binding to synaptosomes isolated from HP of 6 human subjects; data represent the mean ± SD; ***P = 0.0005, 0.0001, 0.0001, 0.0005, and ****P < 0.0001 compared with synaptosomes not challenged with oligomers (biological replicates n = 6; one-way ANOVA plus Dunnett’s multiple comparisons). g) Effect of increasing AβO concentrations (0.5–1–2.5–5–10 µM) on TauO binding to synaptosomes isolated from FC of 5 human subjects; data represent the mean ± SD; ****P < 0.0001 compared with synaptosomes not challenged with oligomers (biological replicates n = 5; one-way ANOVA plus Dunnett’s multiple comparisons). h) Effect of increasing AβO concentrations (0.5–1–2.5–5–10 µM) on TauO binding to synaptosomes isolated from HP of 6 human subjects; data represent the mean ± SD; **P = 0.0021, and ****P < 0.0001 compared with synaptosomes not challenged with oligomers (biological replicates n = 6; one-way ANOVA plus Dunnett’s multiple comparison test).

**Supplementary Figures**

**Supplementary Fig. 1. Flow cytometric characterization of AβO and TauO binding to human synapses.** Representative plots showing the binding of AβO [red fluorescence on the x axis] to synaptosomes isolated from human a) FC and b) HP; representative plots showing the binding of TauO [green fluorescence on the y axis] to synaptosomes isolated from human c) FC and d) HP.

**Supplementary Fig. 2. Characterization of AβO and TauO binding to mouse synaptosomes.**

a, b) Binding of AβO to synaptosomes isolated from mouse a) FC and b) HP (expressed as % of AβO positive synaptosomes); data represent the mean ± SD; **P = 0.0052, ***P = 0.009, and ****P < 0.0001 compared with synaptosomes not challenged with oligomers (biological replicates n = 10; independent experiments n = 6; one-way ANOVA plus Dunnett’s multiple comparisons). c, d) Binding of TauO to synaptosomes isolated from mouse c) FC and d) HP (expressed as % of TauO positive synaptosomes); data represent the mean ± SD; *P = 0.05, **P = 0.0089, ***P = 0.0002, and ****P < 0.0001 compared with synaptosomes not challenged with oligomers (biological replicates n = 10; independent experiments n = 6; one-way ANOVA plus Dunnett’s multiple comparisons). Representative flow cytometric plots showing the binding of AβO [red fluorescence on the x axis] to synaptosomes isolated from mouse e) FC and f) HP; representative plots showing the binding of TauO [green fluorescence on the y axis] to synaptosomes isolated from mouse g) FC and h) HP.

**Supplementary Fig. 3. TauO reduce AβO binding to mouse FC and HP synaptosomes, while AβO increase TauO binding.** a, b) Effect of increasing TauO concentrations (0.5–1–2.5–5–10 µM) on AβO binding to synaptosomes isolated from mouse a) FC and b) HP; data represent the mean ± SD; *P = 0.0154 (Fig.S4b; TauO 2.5 µM) and *P = 0.0291 (Fig.S2b; TauO 5 µM), and **P 0.0065 compared with synaptosomes challenged only with AβO 2.5 µM (biological replicates n = 10; independent experiments n = 6; one-way ANOVA plus Dunnett’s multiple comparison test). c, d) Effect of increasing AβO concentrations (0.5–1–2.5–5–10 µM) on TauO binding to synaptosomes isolated from mouse c) FC and d) HP; Data represent the mean ± SD; ***P = 0.0007, and ****P < 0.0001 compared with synaptosomes challenged only with TauO 2.5 µM (biological replicates n = 10; independent experiments n = 6; one-way ANOVA plus Dunnett’s multiple comparison test).

**Supplementary Fig. 4. Characterization of AβO and TauO binding to human synaptosomes in presence of α-synO.** Effect of increasing α-synO concentrations (0.5–1–2.5–5–10 µM) on AβO binding to synaptosomes isolated from human a) FC and b) HP; data represent the mean ± SD; **P = 0.00012, ***P = 0.0002, and ****P < 0.0001 compared with synaptosomes not challenged with oligomers (biological replicates n = 5; independent experiments n = 3; one-way ANOVA plus Dunnett’s multiple comparisons). Effect of increasing α-synO concentrations (0.5–1–2.5–5–10 µM) on TauO binding to synaptosomes isolated from human c) FC and d) HP; data represent the mean ± SD; *P = 0.0418, 0.0368, 0.0446 compared with synaptosomes not challenged with oligomers (biological replicates n = 6; independent experiments n = 3; one-way ANOVA plus Dunnett’s multiple comparisons).

**Supplementary Fig. 5 Confocal microscopy of mouse primary cortical neurons.**

Representative confocal images of double immunofluorescence staining for MAP2 (gray) and PSD95 (red) in mouse primary cortical neurons treated for 30 min with AβO 2.5 µM (blue) and/or TauO 2.5 µM (green) showing the detrimental effects of oligomers on cell morphology; original magnification 63×, scale bar 20 µm.

**Supplementary Fig.6. Effects of PK pretreatment of human FC and HP synaptosomes on AβO and TauO binding.** The protein components of human synaptosome surface were digested with 1 mg/ml of PK, then the synaptosomes were challenged with increasing concentrations of oligomers, and the resulting binding was detected by flow cytometric analysis. a, b) PK pretreatment effects on AβO (0.5–1–2.5–5–10 µM) binding to human a) FC and b) HP synaptosome; data represent the mean ± SD; *P = 0.0246 (AβO 2.5 µM) and *P = 0.0192 (AβO 5 µM) compared with co­­ntrol synaptosomes (no PK pretreatment) (biological replicates n = 6; independent experiments n = 3; two-way ANOVA plus Tukey’s multiple comparison test). c, d) PK pretreatment effects on TauO (0.5–1–2.5–5–10 µM) binding to human c) FC and d) HP synaptosomes; data represent the mean ± SD; *P = 0.03, **P = 0.0026 (TauO 2.5 µM) and **P = 0.0077 (TauO 5 µM), and ****P < 0.0001 compared with control synaptosomes (no PK pretreatment) (biological replicates n = 6; independent experiments n = 3; two-way ANOVA plus Tukey’s multiple comparison test).

­­

**Supplementary Fig.7**

Representative density plots showing the association between GluA1(green fluorescence in x axis) and Nrx1β (red fluorescence in *y*-axis) in size-gated synaptosomes following extracellular, basal or cLTP treatment in a) FC and b) HP synaptosomes. Fluorescence thresholds (non-specific) were set by staining with secondary antibodies only (lower left quadrant). The increase in the percentage of GluA1^+^/Nrx1β^+^ synaptosomes in the upper right quadrant was used as a measure of cLTP relative to basal conditions.
