# Supplementary figures and images for: Aβ/tau oligomer interplay at human synapses supports shifting therapeutic targets for Alzheimer’s disease
Source: Cell Mol Life Sci. 2022 Apr 4;79(4):222. doi: 10.1007/s00018-022-04255-9 (PMC8979934; doi:10.1007/s00018-022-04255-9)

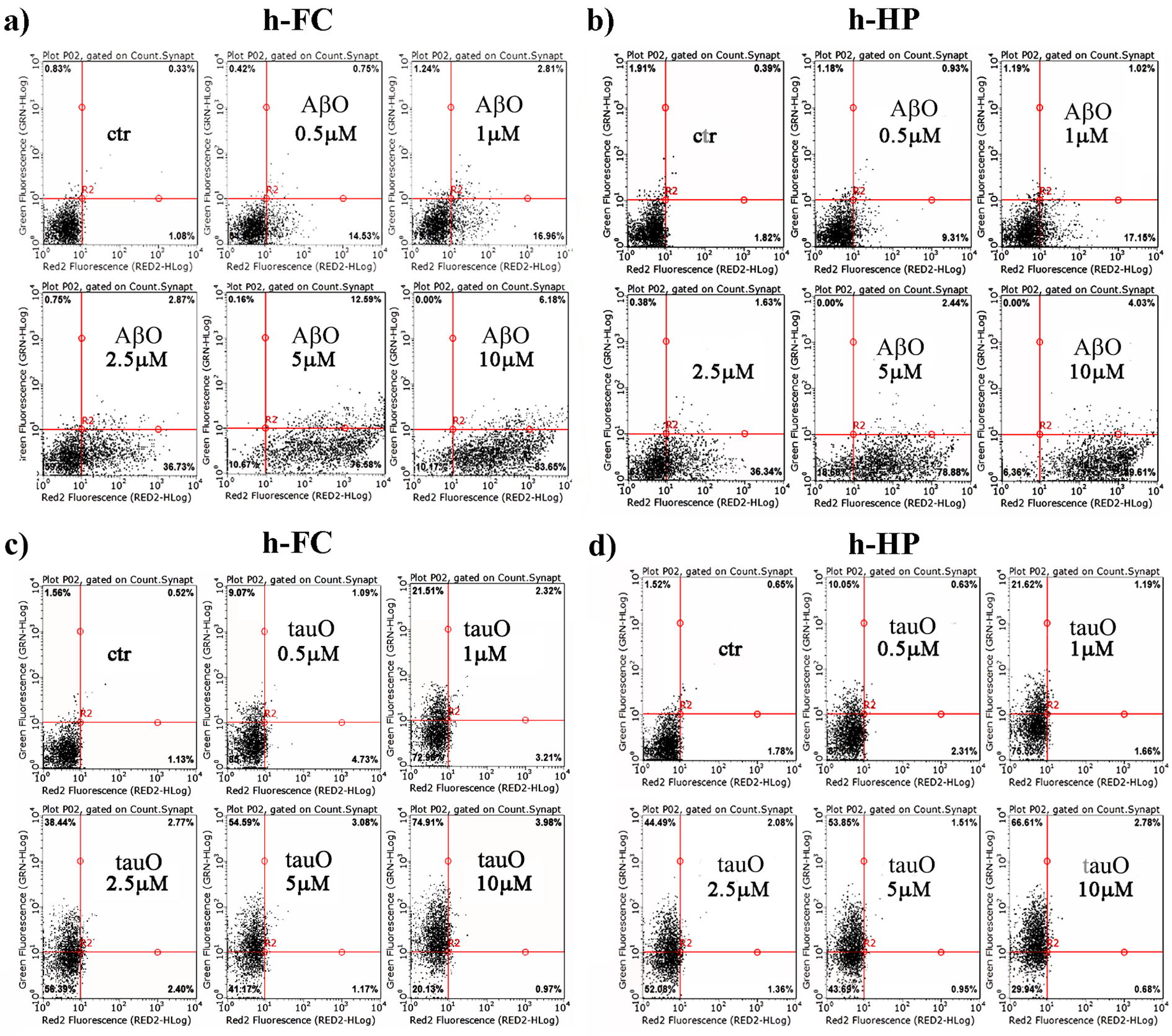

Supplement: Supplementary file 2 — Supplementary file2 (TIF 9288 KB) [file 18_2022_4255_MOESM2_ESM.tif]

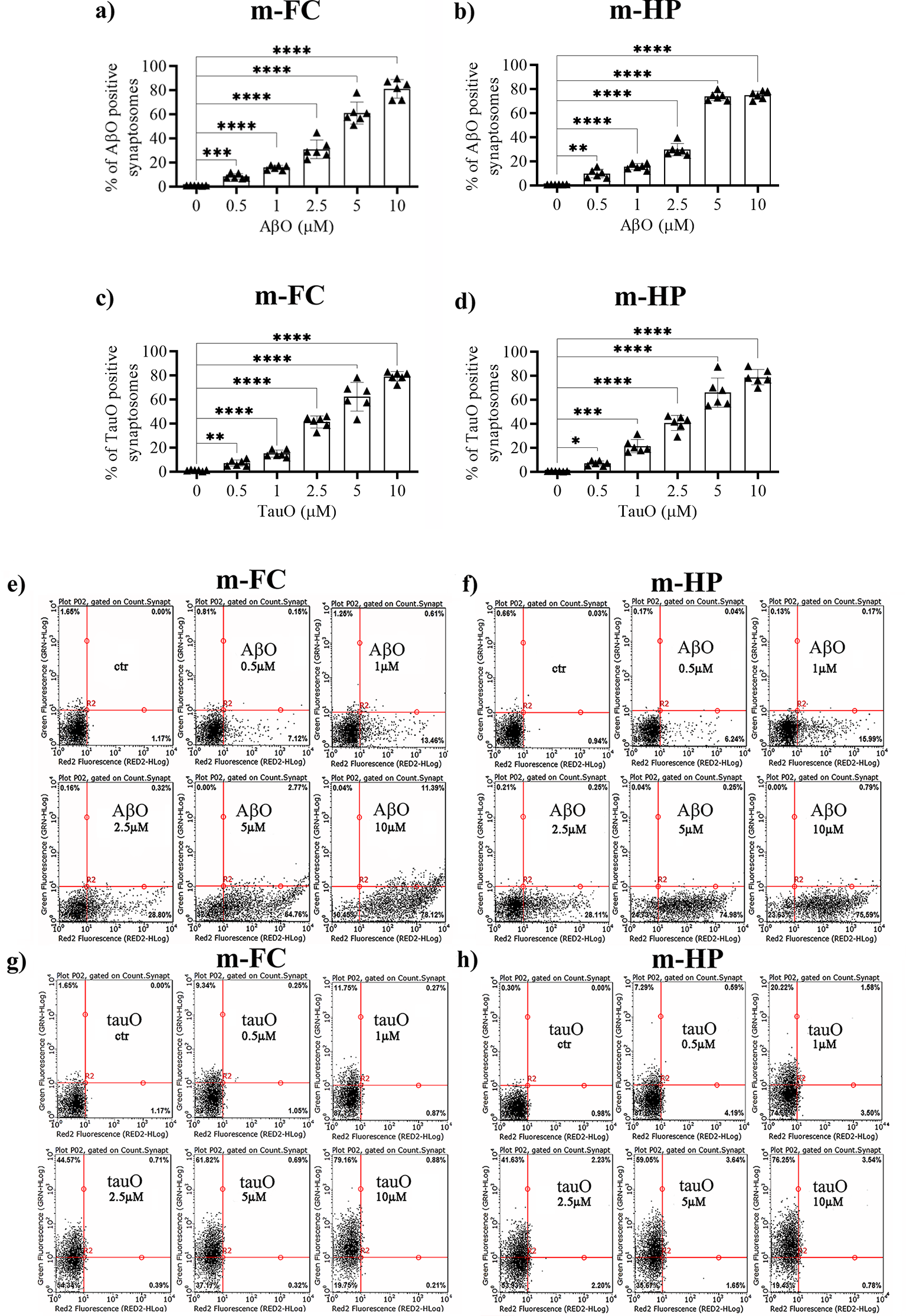

Supplement: Supplementary file 3 — Supplementary file3 (TIF 10064 KB) [file 18_2022_4255_MOESM3_ESM.tif]

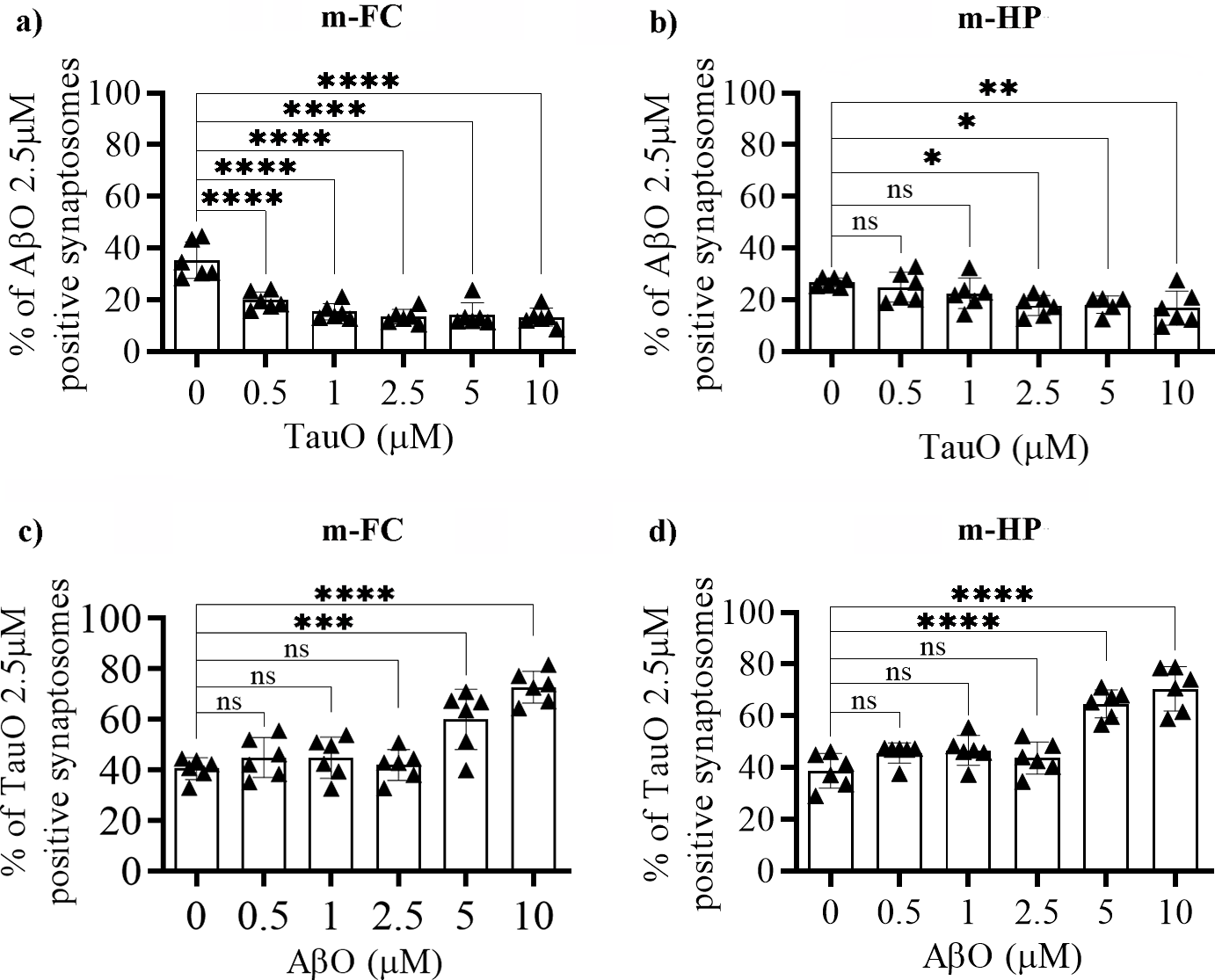

Supplement: Supplementary file 4 — Supplementary file4 (TIF 4552 KB) [file 18_2022_4255_MOESM4_ESM.tif]

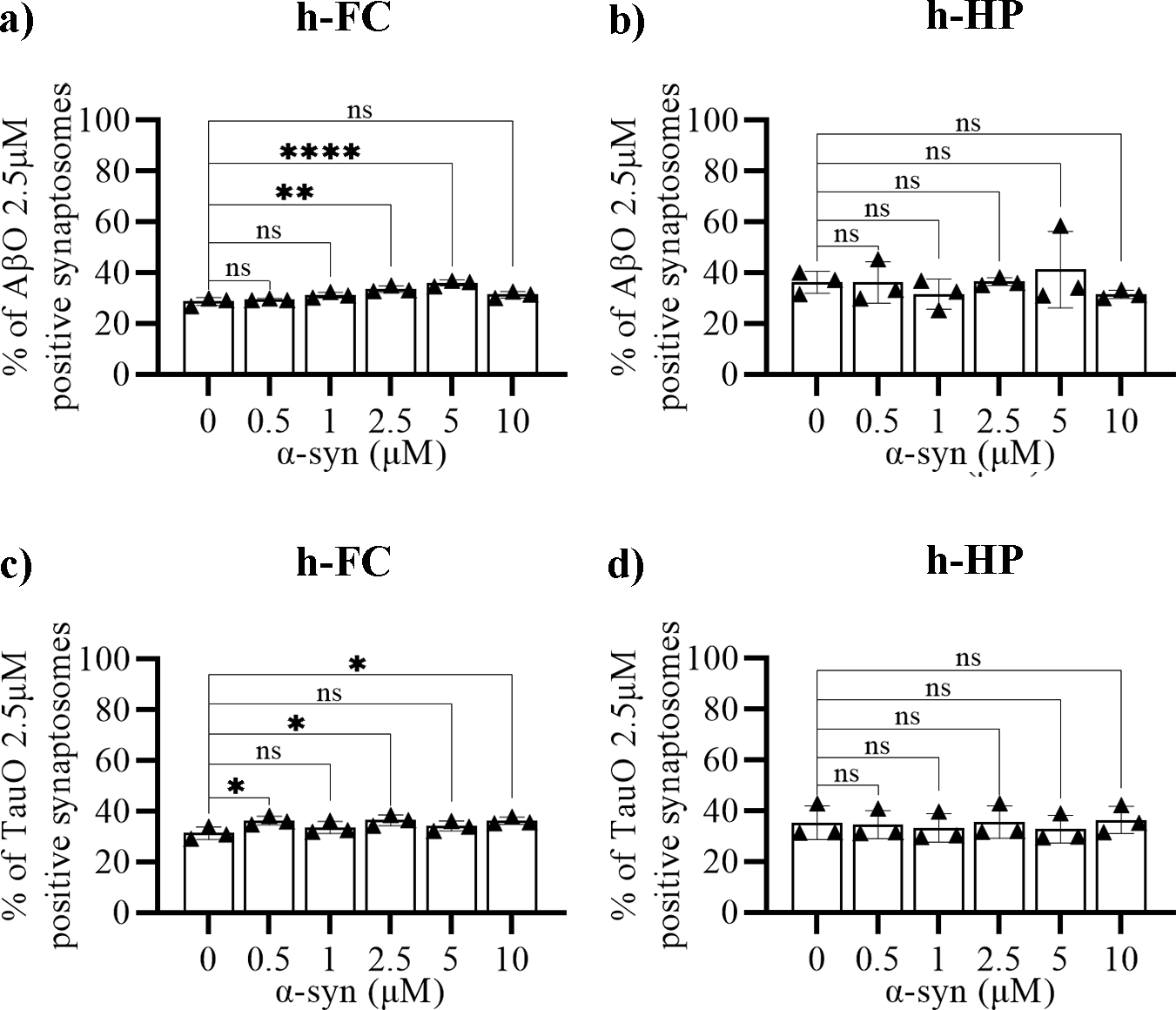

Supplement: Supplementary file 5 — Supplementary file5 (TIF 4628 KB) [file 18_2022_4255_MOESM5_ESM.tif]

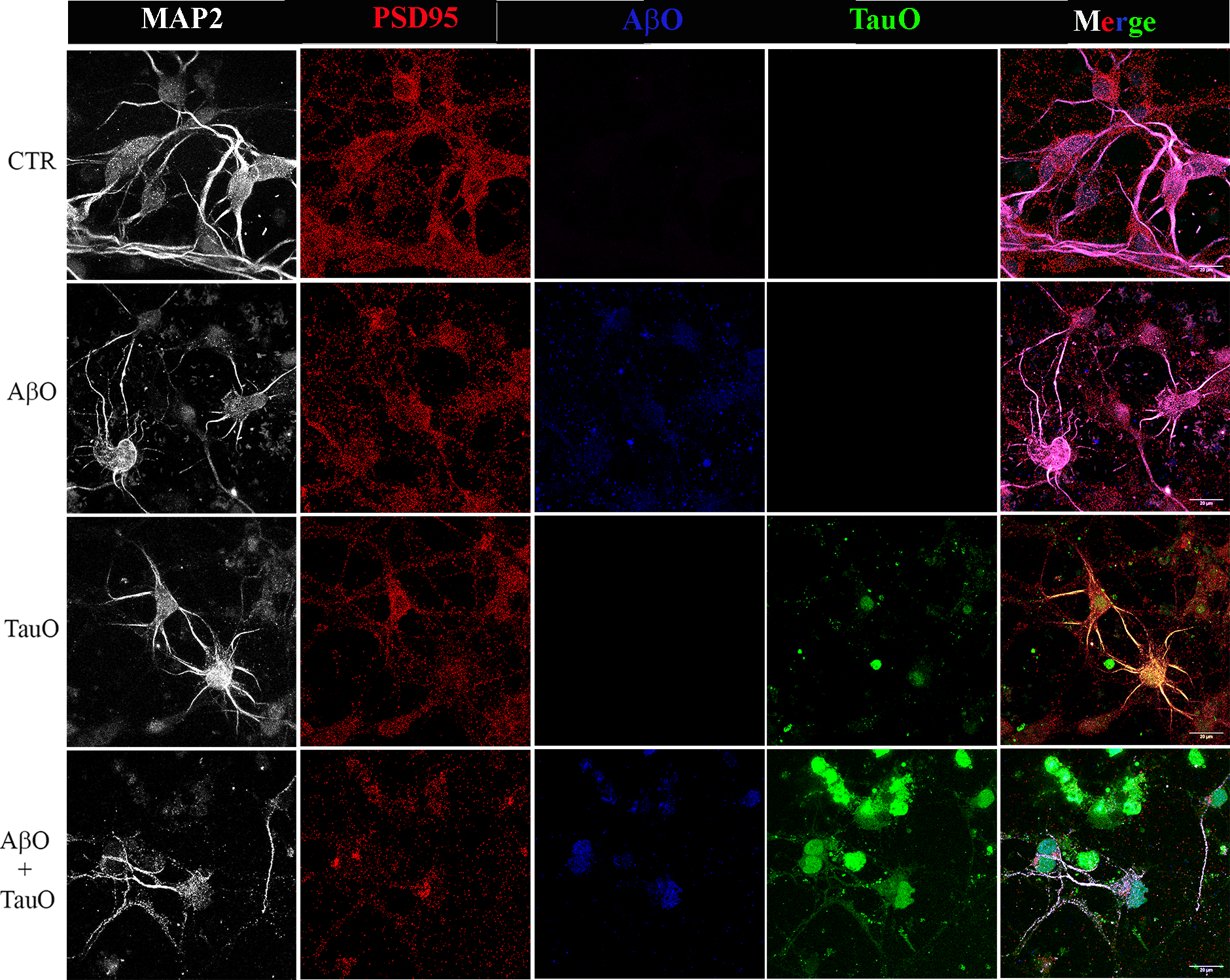

Supplement: Supplementary file 6 — Supplementary file6 (TIF 8280 KB) [file 18_2022_4255_MOESM6_ESM.tif]

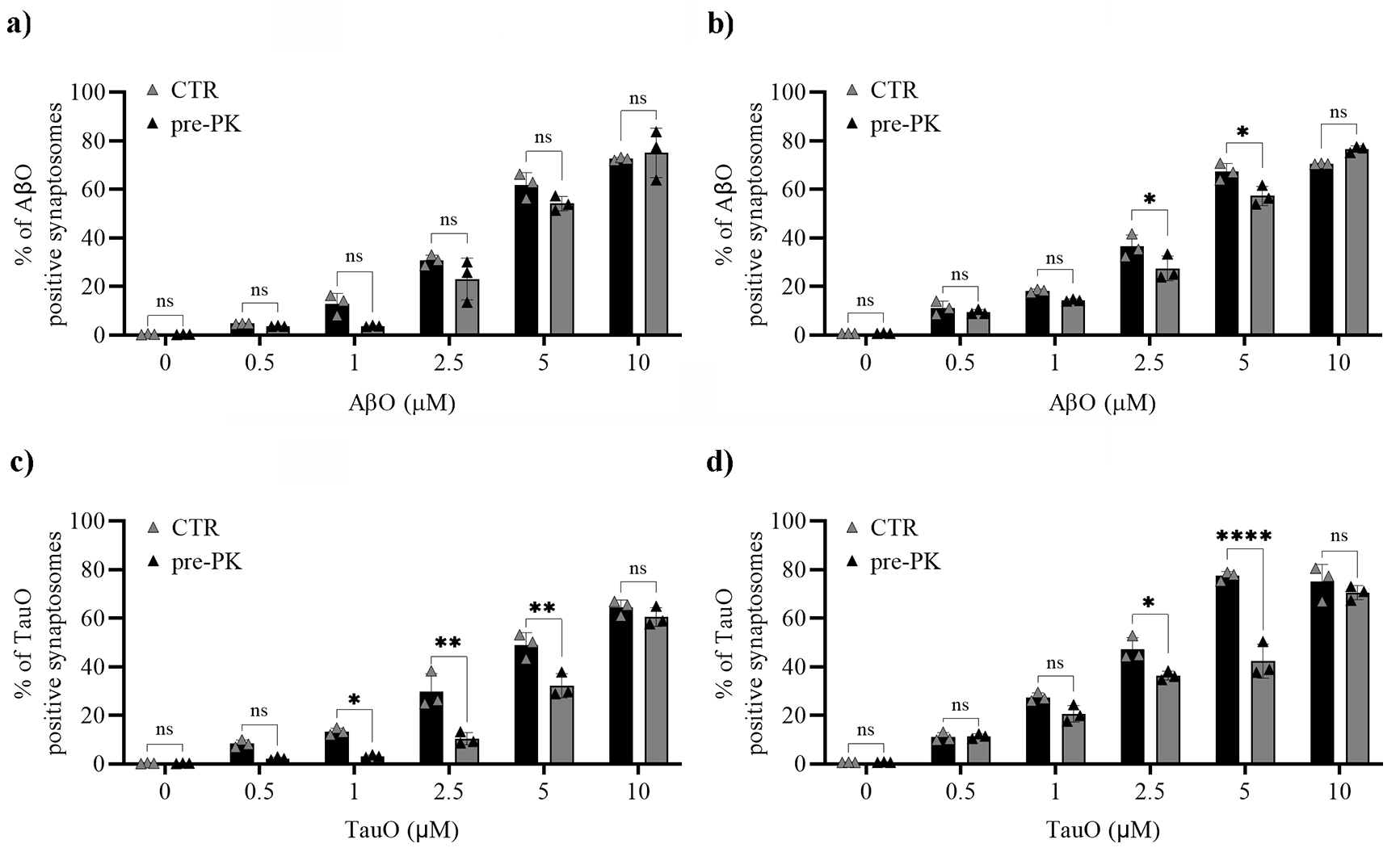

Supplement: Supplementary file 7 — Supplementary file7 (TIF 5390 KB) [file 18_2022_4255_MOESM7_ESM.tif]

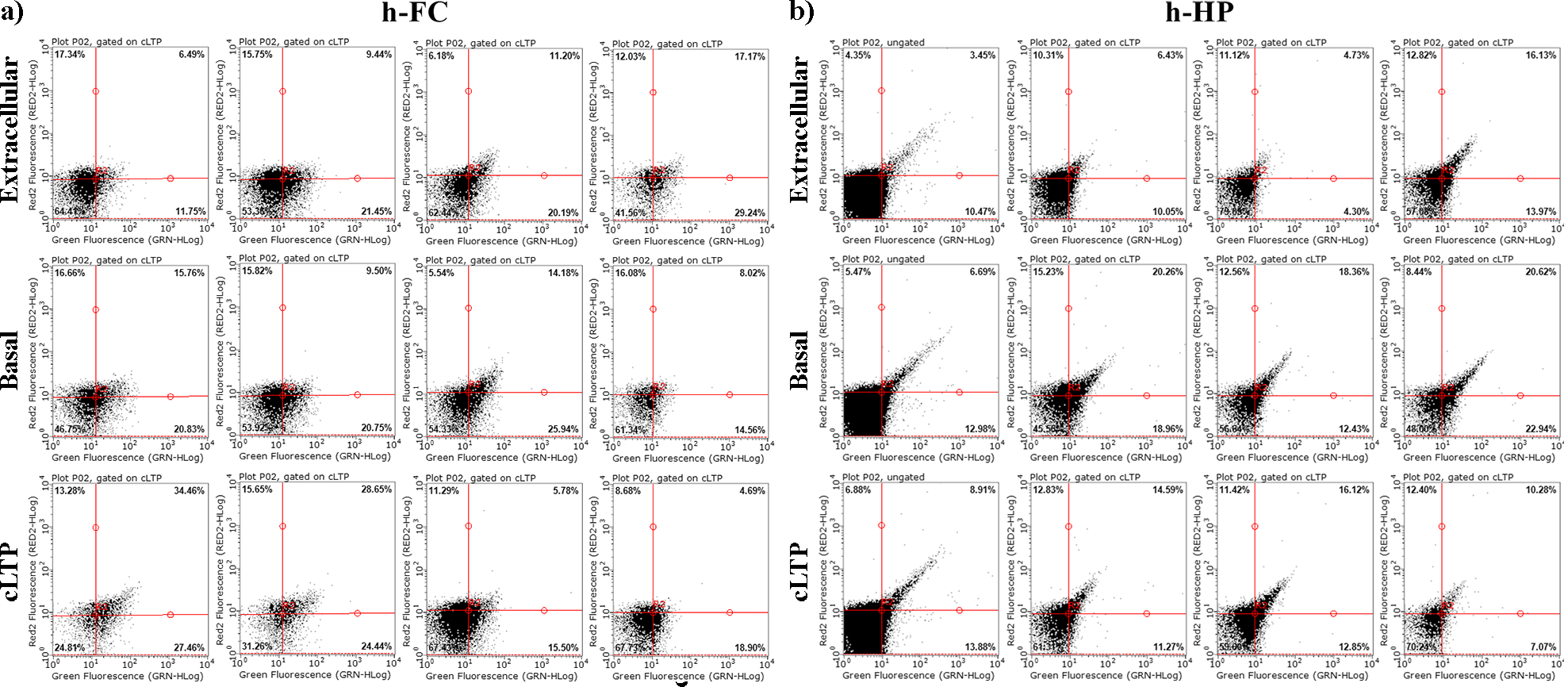

Supplement: Supplementary file 8 — Supplementary file8 (TIF 5041 KB) [file 18_2022_4255_MOESM8_ESM.tif]

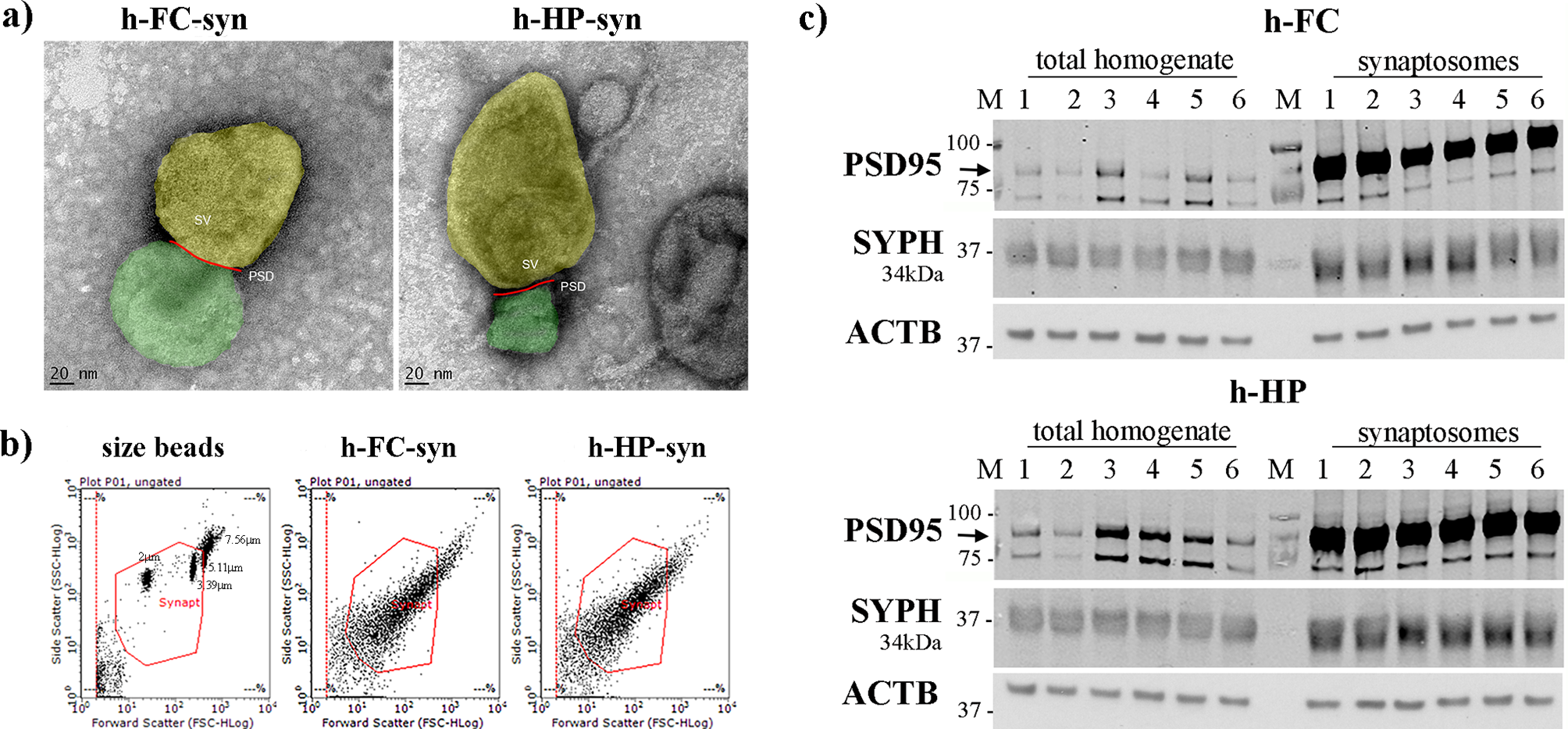

Supplement: Supplementary file 9 — Supplementary file9 (TIF 5515 KB) [file 18_2022_4255_MOESM9_ESM.tif]

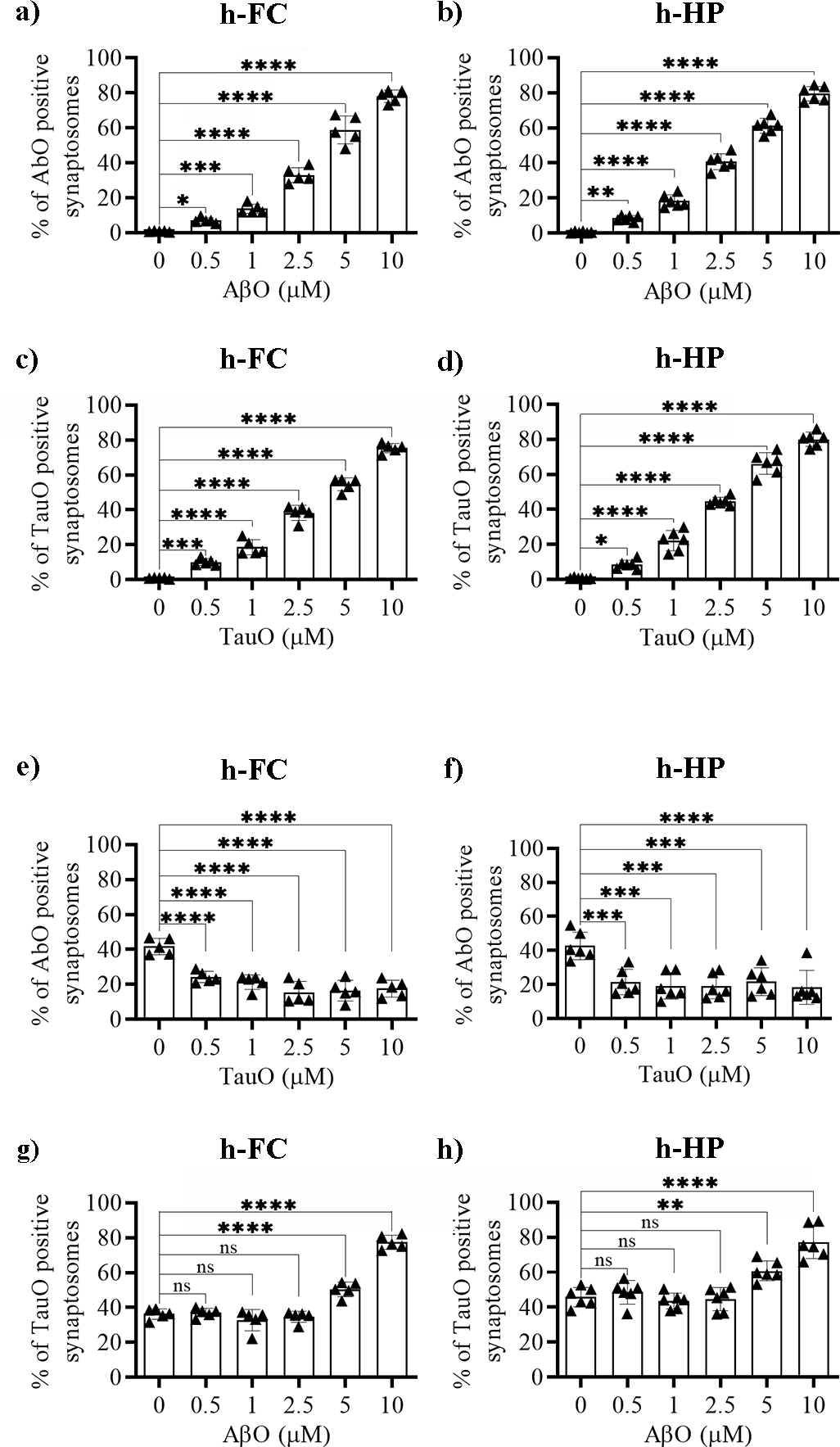

Supplement: Supplementary file 10 — Supplementary file10 (TIF 5348 KB) [file 18_2022_4255_MOESM10_ESM.tif]
